# Botswana tuberculosis (TB) stakeholders broadly support scaling up next-generation whole genome sequencing: Ethical and practical considerations for Botswana and global health

**DOI:** 10.1371/journal.pgph.0002479

**Published:** 2023-11-15

**Authors:** Stephen Molldrem, Sedilame Bagani, Vishnu Subrahmanyam, Rebecca Permar, Ogopotse Matsiri, Cynthia Caiphus, Balladiah Kizito, Chawangwa Modongo, Sanghyuk S. Shin

**Affiliations:** 1 Institute for Bioethics and Health Humanities, University of Texas Medical Branch, Galveston, Texas, United States of America; 2 Victus Global Botswana Organisation, Gaborone, Botswana; 3 Program for Leadership and Character, Office of Academic Advising, Wake Forest University, Winston-Salem, North Carolina, United States of America; 4 Sue & Bill Gross School of Nursing, University of California, Irvine, California, United States of America; The University of Sydney, AUSTRALIA

## Abstract

Global health agencies are increasingly promoting the scale-up of next-generation whole genome sequencing (NG-WGS) of pathogens into infectious disease control programs, including for tuberculosis (TB). However, little is known about how stakeholders in low-to-middle income countries (LMICs) understand the ethics, benefits, and risks of these proposals. We conducted a qualitative study in Greater Gaborone, Botswana to learn how TB stakeholders there viewed a potential scale-up of NG-WGS into Botswana’s TB program. We conducted 30 interviews and four deliberative dialogues with TB stakeholders based in Greater Gaborone, the country’s largest city and capital. We created and showed participants an animated video series about a fictional family that experienced TB diagnosis, treatment, contact tracing, and data uses that were informed by NG-WGS. We analyzed transcripts using reflexive thematic analysis. We found broad support for the scale-up of TB NG-WGS in Botswana, owing to perceived benefits. Support was qualified with statements about ensuring adequate planning, resource-allocation, community and stakeholder engagement, capacity-building, and assessing ethical norms around publishing data. Our results suggest that scaling up NG-WGS for TB in Botswana would be supported by stakeholders there, contingent upon the government and other entities adequately investing in the initiative. These findings are relevant to other LMICs considering scale-ups of NG-WGS and related technologies for infectious diseases and suggest the need for sustained research into the acceptability of pathogen sequencing in other contexts.

## Introduction

Global health funders, the World Health Organization (WHO), regional agencies like the Africa Centres for Disease Control and Prevention, and national ministries of health are increasingly promoting the use of next-generation whole genome sequencing (NG-WGS) and related technologies for infectious disease control, including for *Mycobacterium tuberculosis* (TB) [1, 2]. Pushes to scale up NG-WGS and similar tools such as targeted next-generation sequencing (tNGS) have accelerated following highly visible uses of SARS-CoV-2 genomics during the COVID-19 pandemic, the development of health systems’ sequencing capacities, decreasing sequencing costs, and a growing evidence-base supporting these technologies’ effectiveness [3, 4]. The global scale-up of pathogen genomic surveillance is occurring in “Global North” contexts and low-to-middle-income countries (LMICs) in the “Global South” [5, 6]. However, sequencing technology is complex, costly, and successful implementation requires sustained funding, new socio-technical infrastructures, system-level preparation, ongoing training, as well as an understanding of ethical implications and context-specific challenges [7, 8].

NG-WGS for TB is often promoted because it can reveal details about population-level disease dynamics, drug resistance, “hotspot” areas, groups of people in “clusters” of recent transmission, and other information [6, 9–12]. If properly resourced, TB NG-WGS data can be used for diagnosis, surveillance, and prevention on faster timeframes than other methods such as sample culturing and provides broader information than targeted gene amplification diagnostics (i.e., GeneXpert or tNGS) [7, 13–15].

However, making NG-WGS part of routine practice also comes with risks stemming from the detailed information that the technology can reveal, limited patient and provider understanding, and issues related to cost, trust in health systems, sustainability, and other challenges [7, 16–20]. This is particularly true in LMICs where TB control programs tend to be under-resourced, health systems strained, and where implementations of novel technologies in the name of development have mixed records, including imbrication with ongoing legacies of colonialism and other forms of exploitation [6, 8, 21–24]. Further, attempts to scale-up NG-WGS and uses of pathogen sequence data in research and disease control have caused heated controversies in bioethics and health policy. Examples include the rollout of molecular HIV surveillance in the United States (US), HIV phylogenetics in Southern Africa, and global HIV molecular epidemiology [25–29]. TB is also a stigmatized condition often socially and epidemiologically linked to HIV/AIDS, making it critical to understand social dimensions and ethical issues related to implementation–particularly regarding how NG-WGS data are used in operations and communicated to patients and the public [19, 20].

Against this backdrop, little is known about how stakeholders in LMICs and other contexts understand TB NG-WGS and what their beliefs are about the potential value of integrating NG-WGS into TB programs [18–20]. We thus sought to explore how TB stakeholders in Botswana understood the risks and benefits of NG-WGS as well as the ethical, social, and political issues raised by the potential scale-up of the technology in the country. Our findings are timely, because in July 2023 the WHO issued a communication indicating that tNGS for TB–a technology closely related to NG-WGS–will enter routine standard of care guidelines [3].

## Materials and methods

### Background and approach

This study was conceptualized jointly by the US and Botswana-based team. It emerged from an ongoing TB genomic epidemiology study in Botswana led by CM and SS that began in 2020 stemming from a collaboration started in 2013 [30–34]. CM has also led programmatic TB and HIV work in Botswana since 2008. In 2021, building on the existing study infrastructure, we received supplemental funding to conduct research about bioethical issues involved in uses of NG-WGS for TB research and disease control.

In 2022, we conducted a qualitative study using semi-structured interviews and deliberative dialogues with key TB stakeholders in Greater Gaborone health district, Botswana–which includes the country’s capital and largest city. The recruitment period began on May 2^nd^, 2022, and data collection ended on November 17^th^, 2022. We aimed to understand how participants viewed the benefits, risks, policy challenges, ethical dilemmas, and practical considerations that ought to accompany a scale-up of NG-WGS for TB in Botswana, with relevance for global stakeholders [35–38]. In addition to assessing the acceptability of implementing NG-WGS for TB among stakeholders in Botswana, we sought to “socialize” the technology among participants, educating them about NG-WGS to learn about their understanding of the tool to inform a hypothetical future scale-up [39]. Our design drew on frameworks from Science and Technology Studies (STS), anticipatory governance, and empirical bioethics. We situated our inquiry within the constructivist paradigm of STS, which treats technologies and health interventions not as pre-constituted things, but as complex socio-technical assemblages enacted as much by the functions of a technology as by the socio-political dynamics, people, infrastructures, policy frameworks, and processes that shape implementation in a specific setting [39–41]. From empirical bioethics, we took a two-pronged approach. On the one hand, we foregrounded ethical issues using etic, deductive, or “top-down” frameworks that emphasized bioethical principles (e.g., autonomy, justice, nonmaleficence, beneficence) and ethical concerns related to pathogen genomics regardless of implementation context (e.g., publication, data sharing, consent, revealing transmission dynamics). On the other hand, we also utilized an emic, inductive, or “bottom-up” approach that emphasized participants’ responses and local conditions to identify ethical issues particular to the context in Botswana or which arose from our context but carry more generalizable implications [27, 37, 38, 42]. We drew on anticipatory governance by building our study around what we perceived to be a likely policy future, in which genomic sequencing becomes part of routine TB control in LMICs [6, 26, 35, 36, 43, 44]. Indeed, this anticipated future is somewhat materializing with WHO’s 2023 recommendations for tNGS to become part of routine TB control [3]. While our study focused on stakeholders’ views on NG-WGS, our findings are relevant to tNGS, owing to the technologies’ similar functions (e.g., diagnosing drug resistance, use for hotspot mapping).

### Protection of human subjects and ethics statement

Human subjects research approvals were obtained from the University of California, Irvine Institutional Review Board (IRB), the University of Texas Medical Branch IRB via reliance, and the Botswana Health Research and Development Committee. Participants provided signed consent and were given 50 pulas (~4 USD) per research engagement. Participants could provide consent on forms written in English or Setswana, the official languages spoken in Botswana.

### Participant characteristics, sampling, recruitment, and data collection

Participants were Greater Gaborone-based TB stakeholders purposively sampled and recruited from three key groups: (1) TB policy stakeholders (e.g., from non-governmental organizations, district health management team (DHMTs), and public health officials); (2) TB community stakeholders (e.g., TB survivor-“champions” and other community-based advocates); and (3) TB clinical and research stakeholders (e.g., physicians, nurses, and research staff). Participants were recruited by Victus Global Botswana Organisation (VGBO) staff (CC, SB, and OM) through direct outreach to individuals, organizations, and facilities based on the team’s deep contextual knowledge. Individuals who were enrolled in the parent TB genomic transmission study or who worked for the parent TB study were excluded from recruitment. VGBO is made up of individuals with longstanding ties to the TB care, prevention, and advocacy community in Botswana, and is the host organization for a TB advocacy network. Recruitment outreach included seeking permissions from DHMT offices and related entities, following procedures that are standard in the country, appropriate within Botswana’s regulatory frameworks and cultural norms, and part of our approved protocol [37, 38]. While all participants lived or worked in the Greater Gaborone health district, many had experience working across the country or in roles with a national scope, and our questions were about a hypothetical full-country implementation of TB NG-WGS. Our findings thus provide insight into the views of TB stakeholders in Botswana as a whole, not only those who live in or near the capital.

The study had two branches, with the first involving 30 semi-structured interviews and the second involving four deliberative dialogues with a range of 10 and 22 participants each; the study had 48 total participants (see Table 1). Individuals could participate in either or both branches, including in multiple dialogues. Almost all interviews were conducted by SB at the VGBO offices, in healthcare facilities, and locations suggested by participants (e.g., their offices). Several interviews were conducted virtually. Deliberative dialogues are a focus group-like method designed to allow stakeholders to air out thoughts and feelings about a topic, with the facilitator guiding the group toward articulating areas of consensus and disagreement [45–47]. Each dialogue was structured to bring together different groups within the study population, culminating in the final dialogue with participants from all stakeholder groups (see Table 2). SB facilitated dialogues with assistance from OM, CC, BK, and CM.

**Table 1:** Participant characteristics.

	Number (n = 48)
**TB Stakeholder Category**	
Community	9
Research/Clinical	23
Policy	16
**Branch participated in**	
Interview branch only	14
Interview branch and dialogue branch	16
Dialogue branch only	18
**Botswana citizen?** *	
Yes	42
No	5
**Gender Identity** ^†^	
Female	28
Male	20
**Age** *	
30s	15
40s	17
50s	7
60s or 70s^**^**^	7
**Years living/working in Greater Gaborone** *	
<1–5 years	9
6–10 years	6
More than 10	31

^**^**^Individual variables collapsed together to protect confidentiality because the number who answered for one of the variables was ≤3.

^†^Sex, gender identity, and pronouns were asked, and all participants were cisgender; so, we report these out together.

*Total does not add up to 48 because some participants did not report that characteristic.

**Table 2:** Deliberative dialogue branch structure.

**Dialogue #1, “Community and Research/Clinical Dialogue”**	**(n = 10)**
**Dialogue #2, “Community and Policy Dialogue”**	**(n = 10)**
**Dialogue #3, “Research/Clinical and Policy Dialogue”**	**(n = 13)**
**Dialogue #4, “Concluding Dialogue (All Stakeholder Groups)”**	**(n = 22)**

The total number of participants in the dialogue branch adds up to more than 48 (total number of study participants) because individuals could participate in multiple deliberative dialogues.

Interview guides, deliberative dialogue facilitation guides, and all other study materials were developed collaboratively by US- and Botswana-based team members. Participants could speak in either English or Setswana, with any additional translation assistance provided by the bilingual interviewer (SB) or dialogue facilitation team (SB with assistance from BK, OG, and CM). Interview and deliberative dialogue guides were organized around first assessing participants’ knowledge, and then moving through a discussion of key topics around benefits and risks of the technology such as hotspot and cluster mapping, uses of NG-WGS for TB contact tracing investigations, consent for data uses, data security, the identification of drug resistant types of TB, sharing clinical NG-WGS data hypothetically collected by the national TB program with researchers, as well as transparency and engagement during implementation. The guides are included as (see S1 and S2 Files).

One key feature of our study was an animated video series developed by the team that was shown to participants prior to interviews and dialogues, to educate them about NG-WGS for TB. RP led the conceptualization and creation of the videos, drawing on the fields of graphic medicine and graphic public health [48]. The eight videos told the story of a fictional family in Gaborone that experienced TB diagnosis, treatment, and contact tracing by community health workers (CHWs) that was informed by NG-WGS. The videos also showed scenes of ministry epidemiologists debating whether NG-WGS data ought to be published to show TB hotspot or cluster maps in Gaborone. The full series was shown to participants before interviews. Before dialogues, a condensed version was shown to assembled participants. The videos were designed to help communicate complex details about the information that NG-WGS can reveal, with the aim of opening conversations about ethical issues and implementation challenges.

**Fig 1:**
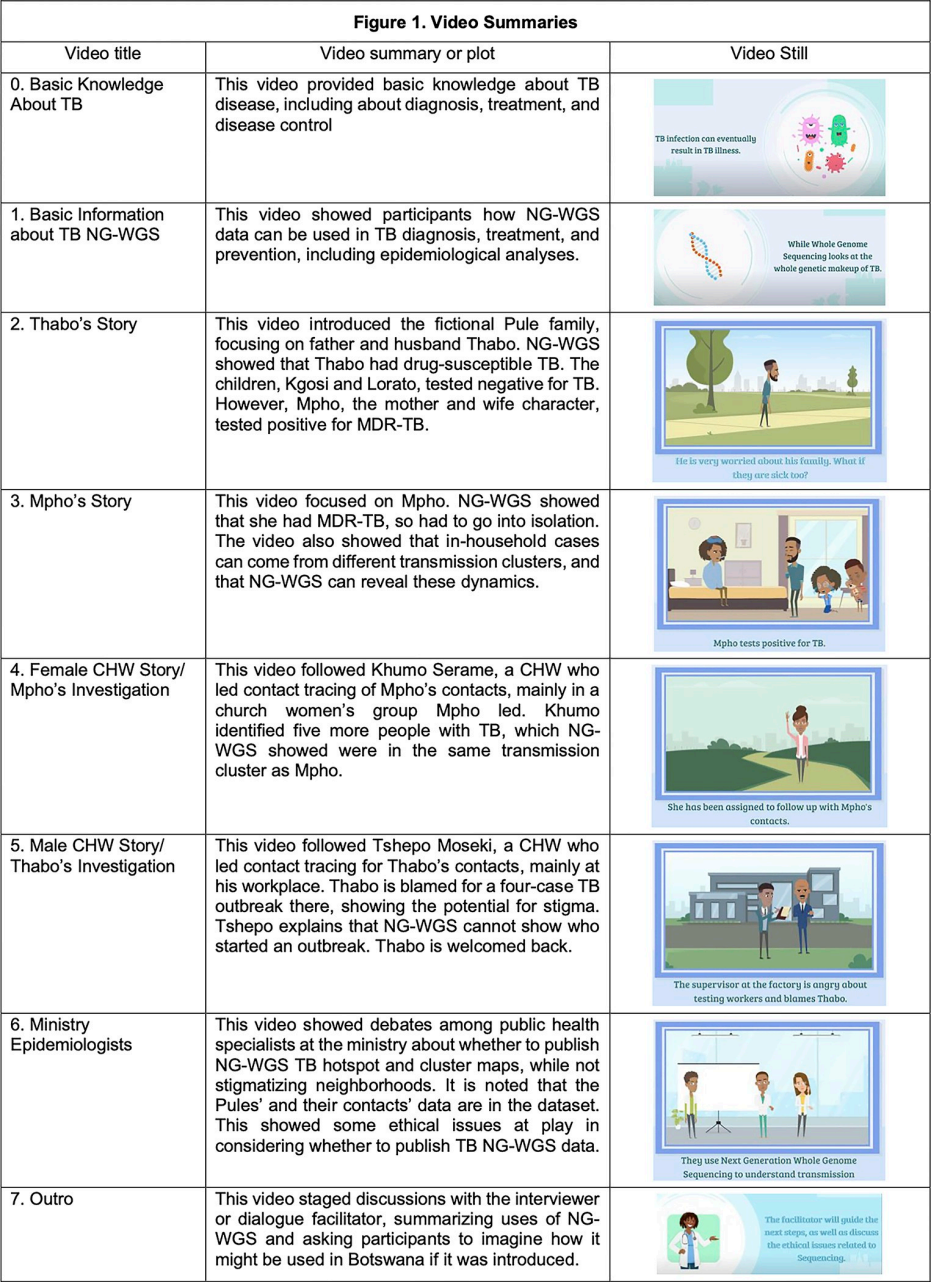
Video summaries.

Rather than providing possible answers or posing questions in a yes/no or either/or style, the videos explained various uses of NG-WGS along with potential ethical issues, and presented situations in which the technology might be used. The aim was to demonstrate potential socio-medical situations and ethical dilemmas that could result from a hypothetical future scale up. For example, in the videos, NG-WGS shows that the husband-and-wife characters are infected with different kinds of TB–drug-susceptible TB and multidrug-resistant TB (MDR-TB)–thus requiring different treatments. Fig 1 below provides brief summaries of the content, purpose, and plot of each video. During development, videos were tested with VGBO personnel for comprehension. Versions in English and Setswana were developed using local voiceover talent. In interviews and dialogues, SB would refer to the videos to guide and enhance discussions with participants; who would also often independently refer to the videos. A methodological paper reporting details about the video production process and participants’ reactions will be published elsewhere, with a focus on implications of our approach for the fields of medical humanities, empirical bioethics, and graphic public health. The video series is provided as (see S1 Text).

The interview phase took place before the deliberative dialogue phase. At the end of interviews, participants were asked if they would be interested in taking part in a dialogue; this recruitment method was part of our approved protocol and a question included on the consent form. Most interviewees were enthusiastic about participation in a dialogue and agreed to be re-contacted. Additional dialogue participants were recruited who had not been interviewed, to ensure that each had an adequate number and range of participants. The dialogues thus involved a mixture of people familiar with the study and individuals who had not been interviewed. This was intentional and led to a variety of perspectives by people with differing levels of familiarity with TB NG-WGS, but who had a common baseline of information provided by the videos. In both interviews and deliberative dialogues, the interviewer and main facilitator (SB) worked to take a neutral position on the question of whether NG-WGS for TB should be scaled up, noting that the purpose of the study was not to advocate for this, but to understand stakeholders’ views.

The process of interviewing participants and talking about interviews and new transcripts in weekly team meetings led to refinements of the interview guide and interviewing approach. Data from the interviews informed the structure of the dialogues. At the start of the fourth and final dialogue, SB showed the videos and presented a slideshow on high-level points of consensus and disagreement about NG-WGS based on data from the interviews and the first three dialogues.

### Data analysis

English transcripts were generated by VGBO staff as data were collected and were analyzed using reflexive thematic analysis in a combined inductive and deductive framework [49, 50]. SM conducted coding using Atlas.Ti. He first generated an initial list of deductive codes driven by known areas of ethical debate or interest in TB genomic epidemiology and topics that came up in the interviews and dialogues. This initial codebook was circulated to the team and further developed by the group over email and during several team meetings. Inductive codes were added iteratively by SM as coding proceeded and were workshopped in team meetings. Interview transcripts were coded first, followed by deliberative dialogue transcripts. Additional deductive codes from Felton et al.–which pertained to rhetorical strategies used in deliberation–were added before coding deliberative dialogues, to better capture consensus, disagreement, and strategies of rhetorical persuasion used by participants [51]. While intercoder reliability is not supported within the qualitative paradigm of reflexive thematic analysis, coded transcripts in Atlas.Ti were shared and discussed with study team members (SB and VS) as coding proceeded, and the process was a recurring topic in weekly team meetings and one-on-one conversations. Discussions about the coding process, the content of interviews and dialogues, and nascent themes were a substantial focus of team meetings during the data collection, data analysis, and writing phases. This process of coding, sharing, and open discussion within the team was the foundation of the reflexive thematic analysis.

The study team also held a dissemination session with TB stakeholders in Gaborone on March 29^th^, 2023, after data collection had ended, coding had concluded, and initial findings had been workshopped within the team. The half-day event involved sharing findings that were precursors to the themes reported in this paper in a slideshow and handout. The brief presentation was followed by full-group discussion about key issues in the study and participants’ thoughts on next steps for TB NG-WGS in Botswana. The conversation involved both English and Setswana discussion, led by SM, SB, OG, BK, and CM. Attendees included participants and other TB and health stakeholders in Botswana. The session was followed by a lunch where conversation continued. While the dissemination session did not involve data collection, feedback from attendees informed ongoing data analysis and future plans.

## Findings

### Botswana TB stakeholders broadly supported scaling up NG-WGS, provided that the country engages in robust planning, capacity-building, and education

Participants voiced their overall support for scaling up NG-WGS for TB in Botswana, owing to many perceived benefits. Benefits frequently discussed included the identification and treatment of drug-resistant TB, revealing transmission hotspots, and assisting in contact tracing investigations by showing clusters. Many participants were very enthusiastic, with one in the second dialogue saying emphatically “Yes, we want it–like, yesterday!”

Similarly, a TB champion (survivor-advocate) spoke to the promise of NG-WGS diagnostics for care and prevention:

*It would make a massive difference because these very people that we help…will be able to access their results much quicker and it would also help them with regard to contact tracing and identifying the people they have been in contact with much faster*. (P01)

There was near-universal support for scaling up TB NG-WGS among participants. However, this support was frequently qualified with assertions that doing so should only take place alongside proper planning, capacity-building, community engagement, and other initiatives to ensure that the technology could be used robustly and sustainably. One TB clinician noted that:

*I think it’s a good test to do nowadays*. *My little concern was with the cost implications*. *But other than that*, *with capacity*, *I think it could actually help for proper diagnosis as mentioned…If there is something that can actually get us on the right track of medications to treat*, *the better*, *rather than doing guesswork*. (P28)

Table 3 provides more statements that reflect the range of reasons and conditions that participants articulated for supporting a scale-up of NG-WGS for TB in Botswana.

**Table 3:** Participants expressed broad-but-conditional support for a scale-up of NG-WGS for TB.

**Near the beginning of the first dialogue, a male TB survivor appealed to his own experience to express support for a scale-up of NG-WGS for TB**: ***TB survivor***: *When I went to the clinic showing signs of TB*, *they took my sputum and I did chest X-ray right*? *And I was enrolled on drug sensitive TB treatment based on symptoms and chest X-ray*. *After a week*, *I then went [back for] culture[-based drug sensitivity testing]*, *and when the test came–I got my results after a very long time–and by the time they came*, *I was drug resistant and not drug sensitive*!*…So*, *I take it that at that time*, *if we had this one*, *of genome sequencing*, *I think that it would have detected quickly the type of TB that I have without having to take a long time*.*‬*
**P07 (nurse and TB coordinator)**: ***Interviewer***: *Is there anything else that you feel has to be considered so that we can eventually arrive at this scenario [an NG-WGS scale-up]*?*‬* ***P07***: *When we are talking about [this]*, *we talk about the cost*, *we talk about the equipment‬*, *about the availability of the catalysts that are being used*. *We talk about the skilled personnel who will be doing the testing*. *I think these are very*, *very important–including the labs*, *because we would have to upgrade our labs*. *Because we have to consider how we are going to store them in these labs*.
**P03 (physician)**: *The advantage is that it’s very rich*. *Let’s start by the knowledge*. *I think I can classify the advantage in two sides*. *Let me start with the professionals*, *including the system*: *Nurses*, *Ministry of Health*, *everyone*, *we [will] have knowledge on something and that is probably new*.*…So*, *it is knowledge*. *And then on the treatment side*, *we may give credit to treatment if we are applying the knowledge that we got and it’s also giving us good results in terms of our cure rate and reduced resistance and so forth*. *This can actually lead to that*. *And also*, *standards*. *I mean*, *I am seeing [other countries] doing it*. *Like*, *we can trace where exactly it is coming from–this particular bacterium that we have in this person*. *So why can’t we reach that standard so we can at least come up with guidelines*? *I know that it might not be very cost-effective for the system*, *but I would want to know how they got to do those tests…There are more advantages compared to disadvantages*.
**P09 (physician):** *Yes*, *so the major benefit is the turnaround time for the results*, *and that is usually quite impactful*, *because…you can’t rely only on the initial testing which shows ‘maybe resistant*.*’ So*, *you need to get the culture and that can take a while although there are other tests which could shorten the time–the lung prognosis*. *Although with the genome sequencing*, *I think the results can be out very fast and that would mean less exposure to the household contacts or just generally any other contact*. *So*, *I think that is a big impact for me*.*‬*
**P25 (District Health Management Team employee)** *I think it’s a step in the right direction–that is*, *if we were to have it*.*‬ It’s really good*, *especially in identifying whether someone is resistant or not resistant*, *and especially the timeline as to when you get the results*. *Because I know that a lot of people that have been treated for TB [with first-line treatment]*, *and it’s not the drug sensitive TB*. *Like*, *you complete the whole six months and then after the six months*, *you develop symptoms again only to find that it was MDR-TB*. *That means that whole time you have already transmitted the resistant TB because it wasn’t treated even though you were on treatment*. *So*, *yeah*, *I think it’s really important and that is a very good example of*, *you know*, *husband and wife–they both have TB*. *Without sequencing*, *we would have assumed that it’s the same type of TB*, *yet it’s not*! *So*, *yeah*, *we really need it*. *I wonder why we don’t have it and I don’t think anyone is thinking about it in our country…I mean in terms of our policy makers*. *I think they should really think about it*.

In sum, TB stakeholders in Botswana expressed broad support for a scale-up of NG-WGS. However, participants also recognized the complexity of this proposal and offered support that was contingent on the country taking a robust and well-considered approach to implementation. Participants’ comments in this regard spanned discussions of costs, proper resourcing, ongoing training, updating guidelines, undertaking community and stakeholder engagement, informing patients about NG-WGS, and a wide range of other issues. In the following themes, we explore how participants discussed these and other topics against the backdrop of their broad support for scaling up NG-WGS for TB in Botswana.

### Efforts to scale up TB NG-WGS must learn from past experiences, such as the introduction of other molecular diagnostics into Botswana’s TB program

When discussing the potential scale-up of NG-WGS, many participants invoked past attempts to improve Botswana’s national TB system with novel diagnostic technologies. They argued that lessons learned from previous initiatives should guide any scale-up of NG-WGS for TB. One reference point that came up repeatedly was the introduction of the GeneXpert molecular diagnostic system in the 2010s, which is used for the diagnosis of TB and rifampin resistance (first-line TB treatment) [34, 52]. While participants spoke about GeneXpert as a general success and as being much faster than culture-based TB drug sensitivity testing, discussions of GeneXpert sometimes referred to lacking funding and resources to ensure its sustainability. Stories about GeneXpert often functioned as lessons to inform a potential NG-WGS scale-up.

One civil society advocate who had previously worked for the national TB program spoke about their ongoing advocacy to resource the GeneXpert technology, which reflects other participants’ comments about the tool:

*I had to lobby with the bosses of the Ministry of Health that ‘[GeneXpert] is going to work but we need money for maintenance*.*’ That is where we have a problem because even now*, *people still call me and say*, *‘the reagents are finished*,*’ you see*…*It was helping people*. *When we went to install it in [a village clinic]*, *people were very happy*. (P05)

While reflecting on a complex clinical case that involved a use of GeneXpert that apparently did not identify drug resistance in a patient, P06 –a nurse and TB focal person–noted that NG-WGS could have been helpful because NG-WGS can identify types of drug resistance that GeneXpert cannot [13, 14]. They said “Before we would use GeneXpert. If it was [after an NG-WGS scale-up] and she came back positive, we could have sequenced and if she came back resistant…we would know that [she had] MDR [multidrug-resistant TB]” (P06). Quotations in Table 4 show how participants invoked the Botswana experience with GeneXpert when discussing an NG-WGS scale-up.

**Table 4:** Participants noted that Botswana’s experience with GeneXpert could inform a potential TB NG-WGS scale-up.

**P17 (civil society stakeholder in community health):** *Obviously*, *[NG-WGS] is not something that comes cheap‬*. *It comes with costs*. *I would relate it to the GeneXpert machines*. *When these were introduced*, *we were battling a little bit as a country in terms of reagents*, *in terms of servicing and maintaining the machines*. *Every district would have these machines*, *but they would not be fully utilized–especially if they were down and not repaired or maintained*, *and when there were no reagents of any sort*. *So*, *this is another thing that we need to be looking into*, *when we are now talking about this technology*. *We need to find what the related costs are*, *what are the benefits*, *because the costs can be high when we start*, *but the return on investments can be quite bigger than the investment costs*. *So*, *we also need to look into whether we have the capacity in the country*, *in terms of utilizing such technology*. *Of course*, *people can be trained*, *but in terms of sustainability in repair and maintenance*, *in terms of supplies that are needed*, *that are used to buy these technologies*, *whether the country will be ready for the that*
**P12 (TB nurse):** *[NG-WGS] can also show the wide range of drugs that the patient is resistant to*, *unlike GeneXpert that just gives us rifampin [resistance data] only*.
**P19 (lab technician):** *Yeah ‘cause right now what happens is at first before they made regulations and guidelines*, *it was up to the clinician to decide which test they are ordering right*? *But now*, *one of the regulations is*, *when a person comes in for diagnosis–especially if it is a fresh diagnosis–it’s automatically GeneXpert first*. *So*, *if the GeneXpert is positive*, *regardless of the rif [rifampin] results*, *it is taken for culture [phenotypic drug sensitivity testing]*. *That’s when other resistances besides the rif will be determined as well*. *So*, *at least with [NG-WGS]*, *that waiting time for culture will be cut down*. *I don’t know*, *because right now we don’t know about the test*. *I don’t know the pros and cons of sequencing versus culture[-based sensitivity testing]*, *because if I knew then I’d know whether it is enough for us to rule out the need for culture or not*. *‬So*, *there*, *I don’t know*. *But*, *if we have this in every testing site*, *it means that GeneXpert won’t be necessary because basically this is technologically better than GeneXpert*, *it’ll be unnecessary*.
**P11 (nurse and TB focal person):** *Is it easily accessible–the expense of acquiring it*, *as compared to GeneXpert*? *I know also that the Xpert machines are expensive*, *but the government has gone ahead and purchased these machines–and we are a step ahead*, *compared to when we were just using [culture-based testing] as our baseline*. *So*, *with this information I think*, *or [even] with this [bioethics] study*, *it helps to enlighten and to bring to light to point out if it is feasible to purchase the [NG-WGS] machines and to do it on a national scale…according to the benefits*, *especially if I’m talking about identifying resistance‬ and identifying drug sensitivity*.
**P05 (civil society advocate):** *I am not saying sequencing is bad*, *what I am trying to say is–like you were saying*, *already other countries [are doing it]*. *They [the ministry] should go there and benchmark*. *Because even for GeneXpert*, *I went to [another country] where they were doing it*, *and I saw it*, *and when I came back*, *there was no time to ask people about GeneXpert–“do you think it’s fine*?*”–because they wouldn’t understand*, *unless you ask a specific group of people like doctors and lab technicians*, *and scientists and so forth…* *People who are experienced with TB sequencing should come and call us and present*, *you know*, *the science behind it*, *so that we can see what is happening*, *right*? *Even with GeneXpert*, *that is what was happening*. *They showed that when you add all the mixtures*, *this is how*, *you know*, *GeneXpert goes through your sputum*.

Partly because of the experience with GeneXpert, participants were acutely aware that if a costly and complex new TB diagnostic technology was introduced into the public health system without adequate resourcing, it might not contribute to a successful response. Scaling up NG-WGS for TB thus appeared contingent upon making these sorts of monetary and logistical investments to ensure long-term sustainability and benefits.

### Public health and research re-uses of TB NG-WGS data were generally supported, even without specific consent, and informing patients about how their data are used was seen as important

Participants were generally supportive of the national TB program using patients’ clinical TB NG-WGS data for routine public health purposes and of sharing de-identified data with researchers for secondary analyses. For most participants, both forms of data use were still supported after it was explained that people receiving TB treatment are not afforded a chance to consent to re-uses of their data for either public health surveillance or retrospective analyses by researchers. Per P06 (a nurse), who held this view, “I don’t have any power [at the ministry]. But I don’t personally see any problem with [the ministry] taking information. This is done in an attempt to improve the situation‬. So, there is no problem…Everyone [who works with TB program data] will have their own code of conduct until the information eventually helps us improve the situation. I don’t believe that there are any major challenges.” Consent was one focus of our study because its relationship to public health practice is an enduring topic in bioethics and has been an area of controversy in molecular HIV surveillance [25, 28, 42, 53]. While lacking consent affordances for routine uses of patient data in TB public health programs were not a concern among Botswana TB stakeholders, some participants did express skepticism about sharing TB program data with researchers without consent. For example, P20 (a civil society advocate) discussed the possibility of introducing “broad consent” into Botswana’s healthcare system to govern future uses of patients’ TB data for both routine public health uses and secondary research purposes, but also noted that broad consent “is quite a new thing” and would pose feasibility challenges.

In dialogue with the issue of consent, numerous participants said that TB patients should be informed about how their clinical data are used by the public health system. Per one TB clinician, “we should put all over the walls of the clinic that ‘in the interest of public health, all data that is collected here is anonymously saved and analyzed to help you‬.’ I think they can be informed by that” (P21). Similarly, P22 –a TB survivor–stated that patients should be informed about how their data are used for public health: “as someone is getting data from me, they should be telling me that my data might be used for this and this and that.” When the interviewer made it clear that public health uses of patients’ TB data and research re-uses are done without specific consent, this participant said that “when things get tough and there is a national crisis, they should use the data; when it’s things that really have to do with a national concern and national security, they should use the data. The Ministry of Health will see how to go about it.” These and other similar statements reflect a general attitude of trust toward state institutions held by most Botswana citizens stemming from effective statecraft and development policies following independence from colonization in 1966 [37, 54–56]. Even so, participants held mixed views about whether people in Botswana trust the country’s healthcare system, with P13, a policy stakeholder in government, saying that “it’s 50–50.”

Support for re-uses of TB patients’ clinical data by public health authorities without specific consent, along with informing patients about how their data could be used, were topically linked areas of consensus. However, as with any consensus, there was not universal agreement. For example, P14 (a public health policy stakeholder in government) took the position that asking patients for their consent for secondary research re-uses of their reportable TB data should be part of the process of enrolling a patient into care, but that routine public health uses of patients’ clinical TB data should not require consent. P14 noted their belief that retrospective studies of public health data by researchers were “not the same as we do [at the ministry]” in TB disease control. When asked about potential data quality ramifications of requiring TB patients to give specific consent for secondary research re-uses of their public health data, P14 argued that ‬‬“if you see a large number of people refusing, that means we need to improve our education system–that means people are not educated and they are not aware of the benefits of submitting that data‬.”‬‬‬‬‬‬‬‬‬‬‬‬‬‬‬‬‬‬‬‬‬‬‬‬‬

Implicit in P14’s position is that patients would give consent to secondary research re-uses of their reportable public health data if benefits to society were explained to them. While that position would need to be verified through further studies, P14’s support for patient education is aligned with our overall finding that informing patients about how their TB data could be used for public health programs and research purposes was supported across our sample. Table 5 shows the varied views participants held about how, where, and when Botswana’s health system should seek consent or educate patients about uses of their data.

**Table 5:** Participants supported public health uses of TB data without specific consent, with some reservations.

**P01 (TB survivor-advocate), when asked about consent, public health, and publication:** ***P01***: *For as long as they don’t write my name*.*‬* ***Interviewer***: *So*, *your main concern is on the name*? ***P01***: *Yes*. *For as long as there is privacy*, *I don’t think there is a problem*. *I mean*, *as long as there is no identification pointing towards us*. ***Interviewer***: *Even if you didn’t give consent*, *for as long as they don’t include your name*? ***P01***: *Yes*! *Privacy*! *For as long as they don’t include names*. ***Interviewer***: *OK*. *I hear you and understand you very well*.
**P03 (physician):** ***P03***: *Within the public health system*, *we will have trends and those trends are being studied based on the samples that we have*.*‬ So those are things that we always use*. *I don’t think it should be compromising–it shouldn’t compromise anything*. *But*, *in the beginning*, *if that sample was taken and then the person signed a consent for it*, *that’s where the problem comes when you want to re-use [for research] without a consent*. ***Interviewer***: *Oh yeah*, *definitely*! ***P03***: *You understand*? ***Interviewer***: *Research is an entirely different thing*. ***P03***: *Definitely*. *Whenever we give consents in the public*, *we have a book where when someone has an infectious disease like malaria*, *tick*, *and whatever you can think about–we use those for public health purpose*. *We don’t need to consent from the patient*, *because it’s what we see and we cannot give anyone to consent about it*. ***Interviewer***: *Yeah*! *That’s exactly what I am saying*.*‬* ***P03***: *As long as people can re-use it but initially if someone came to me and signed a consent form for HIV test for whatever*, *I wouldn’t really be comfortable having people re-using it to publish and so forth*. ***Interviewer***: *So as long as it is used like within to help with TB control efforts and all of that-for the good of the public*, *then it is fine*? ***P03***: *Mhm*.
**P04 (policy stakeholder):** ***P04***: *[Routinely collected clinical data] is the data that is informing our policies as a nation…So*, *right now*, *currently*, *the Ministry is trying to build a hub where data will be accessible to those who want to use it [e*.*g*., *researchers]–but*, *as the Ministry and on behalf of those patients and everyone we have collected data from*. *That is why we always have a research unit and when you come to a certain program to say*, *‘I have been given authority to collect or to use this data*,*’ you still need a letter and that letter on its own is the one that is trying to advocate for the government*. *‬*‬‬‬‬‬‬‬‬‬‬‬‬‬‬‬‬‬‬‬‬‬‬‬‬‬‬ *So*, *if it is [university]*, *that has given you the permission to go and analyze TB or the retrospective TB data on this and this*, *we need that documentation*. *And the*, *in fact*, *we have a lot of paperwork*. *The files are this big [gestures]*. *So*, *those that are researchers*, *they come*, *they collect data*, *we sit down with them and guide them–but with the authority of the Permanent Secretary of course*. ***Interviewer***: *So*, *this is something that you would likely keep ongoing even when sequencing is introduced in public health*? ***P04***: *Yes*. ***Interviewer***: *Like you don’t see that changing in any way*? ***P04***: *Yes*. *I can just imagine–if you were to ask for a consent from each and every one*, *our interventions will never work…[But]*, *awareness is something that is very important*. *It should be there*.*‬*
**P16 (community health worker):** ***Interviewer***: *So*, *you feel that there is no issue with the lack of consent from the owners of the information because the information is created to help‬*? ***P16***: Akere *[isn’t it]*, *we won’t be sharing anyone’s names*. *They won’t know who is who*. ***Interviewer***: *Mhm* ***P16***: *I feel like it doesn’t really matter as long as their names are kept hidden*. *There are no names even in our annual reports*.
**P02 (research project manager)**: ***Interviewer***: *But we both agree that when Thabo gave a sputum sample*, *he did not consent [for analyses or publication of his data]*?*‬* ***P02***: *Mhm*. ***Interviewer***: *So*, *according to you*, *do you feel that for as long as it is used appropriately and it is given [to researchers] on request*, *and there is no information on Thabo that identifies him*, *then it can be shared*? ***P02***: *It can be shared*. ***Interviewer***: *Oh*, *OK*. *In the public health interest so that it helps in controlling TB*? ***P02***: *Yes*! *Because it can even bring other things that another person did not think of*. *Isn’t it that when researchers are there*, *they think of other things right*? *So*, *that information can be handy in that case*, *instead of going back to start afresh [to gather new data]*. *So*, *we just take the one that is available [at the ministry] which will benefit the whole nation at large*.

In sum, statements by participants reflected a consensus that public health uses of individuals’ clinical data often do not allow for specific consent. This view generally extended to secondary uses of de-identified TB program data for retrospective research, but not for all participants. One remedy offered for lacking consent affordances was that information be given to TB patients about how their clinical data could be used to support Botswana’s TB response.

### Ongoing community and stakeholder engagement along with education for patients, healthcare staff, lab workers, and the public should be part of any TB NG-WGS scale-up

Participants stressed that community and stakeholder engagement–along with educational programs aimed at the TB workforce and the public–should be central to any scale-up of TB NG-WGS in Botswana. These efforts were seen as initiatives that should begin prior to scale-up, continue during implementation, and then become ongoing. There was consensus that education and engagement ought not be limited to consultations prior to implementation or merely aimed at securing stakeholders’ support. Rather, these efforts should be central activities and seen as crucial to successful implementation.

One participant who worked in a lab that handles TB samples emphasized the need for health workforce training. They said “Obviously, it is a new thing…so people will have to be thoroughly trained. Not just the testing staff but everyone involved, because there is a new kid on the block. So, introduce it” (P19). P02, a research staff person, also noted that clinical staff “have to be taught; we shouldn’t take it for granted just because they are health care workers‬‬–‘so, sequencing is here!’–then we just stop there…Let’s train them so that they know what sequencing is, how is it going to benefit patients, and how should they be working around it as health care workers so that once it comes, they are prepared.‬” P26, a TB program coordinator and nurse, connected the issue of health workforce education for NG-WGS to patient rights and education:‬‬‬‬‬‬‬‬‬‬‬‬‬‬‬‬‬‬‬‬‬‬‬‬‬‬‬‬‬‬‬‬‬‬‬‬‬‬‬‬‬‬‬‬‬‬‬‬‬‬‬

*Health care workers are also ignorant about the rights of the patients*. *We need to teach them faster*. *There is community TB care in the guidelines; it has the patient charter in there–the rights*, *right to privacy*, *right to information*, *right to confidentiality*, *etc*.*…Those should also be emphasized for each and every patient who starts on treatment*: *being taught about TB*, *the basics of TB*, *counselling should be given to them about expectations from the day of diagnosis until the end of treatment*.*‬*‬

Participants also emphasized that educational efforts ought to include sustained and coordinated public communications, community engagement, and stakeholder engagement. A statement by one TB survivor-advocate captures these overall sentiments:

*I think [the government] should contact the relevant stakeholders…the affected communities*, *the community leaders*, *the chiefs [*kgosis*]*, *and parliamentarians*. *They have to spread the message and inform us about the technology they will be introducing*. *So*, *we have to engage the parliamentarians*, *call* kgotla *meetings and inform the public of the government’s intention to introduce such a technology–to explain to them that they have been used in detecting COVID-19 but want to now start using it for tuberculosis…‬‬‬*‬‬‬‬‬‬‬‬‬‬‬‬‬‬‬‬‬‬‬‬‬‬‬‬‬‬‬‬‬‬‬‬‬‬‬‬‬‬‬‬‬‬‬‬‬‬‬‬‬‬‬‬‬

*They should consult stakeholders…spread the message through radio or social media so that this message can reach a larger population…engage journalists*, *the print media*. *They should be taught about this thing and be sensitized about it and then publish articles in their newspapers*. *I think that could also help*. (P01)

Other comments about public education and communication included anti-TB stigma messaging and working with existing civil society organizations to enhance government-led efforts. Two participants in the first dialogue suggested forming a new civil society organization that would play a role in education related to a scale-up of NG-WGS. One noted that “as a civil society person, I would advise the TB survivors or the professionals to form a very‬, very strong advocacy group so that we can speak with one voice‬.‬” When discussing the logistics of a scale-up, P07, a TB nurse and program coordinator, noted that “I think even the community could be informed; they can be informed that TB nowadays is tested this way. Those are the resources I would need to equip for training, for facilitation, and to teach people.”‬ Table 6 shows other statements by participants emphasizing the need for ongoing forms of public engagement and patient education, including engaging *kgotla* meetings (traditional community gatherings designed to support local governance and decision-making) and *kgosi*s (hereditary chiefs).‬‬‬‬‬‬‬‬‬‬‬‬‬‬‬‬‬‬‬‬‬‬‬‬‬

**Table 6:** Participants advocated for ongoing engagement and education about TB NG-WGS in the event of a scale-up.

**P27 (physician)**: *That also means taking time to actually educate the patients–or should I say the public–and that could be*, *like*, *I think working through our traditional system of the* kgotlas, *you know the public*, *through the* kgotlas, *you know all these resources*: kgotlas, *churches*, *and all that*, *so that there is information out there and people have the chance to ask questions and to get the answers they need*. *Because it shouldn’t be just in the clinics*, *I think*, *so that when they come to the clinic there is already some kind of understanding of what this whole thing is all about*. *So*, *we would have to invest in this*, *yeah*. *Public health campaigns*, *public education*, *yeah*.*‬*..*You’ve got to get the buy in of your gatekeepers right*, *you’ve got to get the buy in of the* kgosis *(chiefs) and all these development committees*, *the church leaders and that sort of thing*. *Get buy-in from them so that they can be part of it*. *If they can understand*, *then we can just move smoothly I think*.
**An exchange about community engagement and a scale-up of NG-WGS for TB between the interviewer and P09 (a physician):** ***P09***: *I’ve never thought about this*, *but I believe that for the success of anything people need to be prepared*.*‬ In terms of policies*, *sometimes [they] occur at higher levels and they are passed down and just implemented top-down*. *But I think the approach which we neglect is bottom-up*, *whereby you actually hear from the people first and not necessarily people like me who are the caretakers–we are talking of the end users*, *the patients themselves…So we hear from them and then inform*. *I don’t know whether these policies are guided in that way*. ***Interviewer***: *So*, *that is your recommendation–to ask the community*? ***P09***: *That would be a good recommendation*. *It would be a good approach*, *especially when we talk about public health*. *If it is the health of the public*, *we need to get their input as well*.
**In the fourth dialogue, two participants discussed possible partnerships and education across programs, noting that sequencing is done for HIV in Botswana, but not TB:** ***Male 1***: *Somebody was talking about the education that we should have–that we should educate the community about the benefits or the risks of this test kit*. *We should do it*, *I think*. *Now it’s time for us as TB survivors to assist in that*. *We have got the experience*. *So*, *I am asking from people who are in the National TB Program who are here to consider that*. *When you start implementing things*, *remember us so that we can do community awareness and tell people about the benefits of whole genome sequencing of TB*.*‬*‬‬‬‬‬‬‬‬‬‬‬‬‬‬‬‬‬‬‬‬‬‬‬‬‬‬ ***Facilitator***: *[Calls on next speaker] Yes*! ***Female 5***: *I just want to say that I think it’s important also*, *because I am more on the HIV side*. *HIV is doing this*, *and HIV is going to keep doing this–right or wrong–we are going to need to actively discuss it*. *Because that’s the way that technology is going towards with regard to patient care*, *so it’s kind of a done deal*. *But I don’t think TB should hide in there*, *it should come together with HIV*. *We should see how we can do these things together and collaborate and start the lessons…Because HIV is already well established in whole genome sequencing*. *So*, *I just want to say that from the ethical point of view*, *we shouldn’t let TB lag behind where HIV is already doing or is already established*.*‬*

Broad and ongoing stakeholder engagement, patient education, and healthcare workforce education were roundly supported. This reflects emerging best practices in global and community-based health practice. However, participants’ recommendations also reflect the distinctive makeup of Botswana’s political system, which combines elements of traditional culture such as *kgosis*, *kgotla* meetings, and generally-trusted state institutions, including a comparatively robust healthcare system [37, 54, 55]. These entities, along with media organizations and advocacy groups, were all seen as playing different-but-vital roles in an NG-WGS scale-up.

### Scaling up TB NG-WGS in Botswana should involve deliberations about ethical norms for publishing data, particularly regarding transmission hotspots, clusters, and stigmatized groups disproportionately impacted by TB

There was general agreement that introducing NG-WGS into Botswana’s TB program should be accompanied by assessing some of the ethical norms that govern how the country uses TB public health data. Participants spoke about this regarding data uses for disease control, such as for diagnosis and contact tracing. However, issues about ethical data use were most pronounced around how TB NG-WGS data ought to be published. P22 (a TB survivor) noted that widely disseminating TB NG-WGS data might not be beneficial and could “instill fear in people.” They then said that “maybe [the Ministry of Health] can just tell the part of the city that is affected. Maybe they should just go to the *kgotla* and inform them, ask them to do tests and sequencing and what not‬.” Others supported widely publishing data, with P16 (a community health worker) noting that “I think that if the community is aware, they can also help fight the disease, because if we try to keep certain things secret, things will only get worse‬.” In sum, participants acknowledged the need for new ethical norms regarding the publication and dissemination of TB NG-WGS data; however, no clear position emerged about what those norms ought to be.‬‬‬‬‬‬‬‬‬‬‬‬‬‬‬‬‬‬‬‬‬‬‬‬‬‬‬‬‬‬‬‬‬‬‬

To this point, there was a lack of consensus regarding the ethics of publication practices regarding TB NG-WGS *hotspot maps* and *cluster maps*, two common ways of displaying genomic TB data that were shown to participants in the animated videos. Hotspot maps generally display geographic areas where transmission is concentrated (e.g., a city district), whereas cluster maps show networks of individual people represented by dots connected to each other by lines showing molecular or non-molecular (i.e., behavioral) epidemiological connections.

Some participants did not see clear ethical distinctions between these different ways of displaying TB genomic data, whereas others saw cluster maps as being higher risk for potentially identifying individuals. There were also concerns that publishing either kind of map could stigmatize neighborhoods or subgroups affected by TB that are already disenfranchised by further associating them with TB (e.g., poor districts, migrant workers, people living with HIV). P17, who worked in civil society and community healthcare, said that sensitive TB NG-WGS data about subgroups should “only be shared to platforms at such levels as the ministry…but not necessarily to the community, [kept] in the space of healthcare workers‬.” P17 took this position “so that we can have specific interventions that target certain groups of people, whether they are sex workers, whether its [men who have sex with men], whether its drug users,” and that “it would be ok to share this information within the health space, for those that are going to be assisting in programming. But in terms of [sharing information with] the community, it can bring about some discrimination.‬”‬‬‬‬‬‬‬‬‬‬‬‬‬‬‬‬‬‬‬‬‬‬‬‬‬‬‬

Others saw benefits to broadly publishing TB NG-WGS data, arguing that doing so could contribute to TB prevention education. Per one TB policy stakeholder who supported the publication of hotspot and cluster maps:

*If that area has been labelled as a TB hotspot*, *and those people really know the modes of transmission of TB*, *and they are taught how to prevent from contracting TB*, *then they are going to practice whatever it is that is needed to prevent TB infection*. *So*, *it will not be stigmatization…of course the risks are not outweighed by the advantages*. (P14)

On the other hand, P08 (a TB clinician) had a mixed view, noting that “Information is empowering, right? Information is important, decisions should be made with all possible information.” P08 then added that “information can also breed biases and negativity and unwanted, unforeseen consequences…You don’t mean for people to judge you or your area for being a hotspot.” Further, in the words of one participant in the first dialogue who advocated sharing data with “community leaders” and “champions” before deciding whether to share specific maps publicly: “I always say that when it comes to sharing, do so with caution because for some, you are chasing away their people.‬”

Table 7 shows other participants’ reflections and statements about potential benefits or pitfalls of publishing TB NG-WGS data that could highlight disproportionately affected areas or populations. The quotations show the ambivalence of participants on the question of when or how hotspot or cluster data ought to be shared, and their rationalizations. The exchange presented at the bottom from the second dialogue dramatizes the extent of participants’ mixed and uncertain positions about the benefits of publishing cluster maps.

**Table 7:** Participants debated the ethics of publishing TB NG-WGS data.

**Statement by a male participant during the second deliberative dialogue:** *Like she [another dialogue participant] is saying*, *sharing information is very important*. *My issue is*: *how do we share it*, *to whom*, *and what are we sharing*? *These are the critical things that we have to consider*. *I still look at that one to say*, *‘you spoke about clusters and hotspots of which last time*, *I was made to believe that they are two different things–clusters and hotspots*.*’ So*, *with clusters I believe that*, *for example*, *[names a district in a city]–I mean just my thinking*, *TB is most prevalent in [that area]*. *I don’t see any problem in [sharing] that*. *It’s important to make us aware and with awareness we take precaution*. *It’s very important*. *My issue was with hotspots is the one that covers [a large area] right*? *I want the one that goes in-depth where it says ‘[specific district] we have this [kind of TB]*, *but this kind is the one that is more prevalent and making the disease of this kind spread*.*’ Like*, *that one*, *I feel exposes a lot*. *That’s the one that*, *if we are not careful with it*, *can cause stigma–and stigma is something that we don’t want to be there because if it is there*, *you will find that people will become reluctant and they won’t even come test for TB*. *When they start coughing*, *they would rather lose weight until they die*, *because of fear*.
**P18 (civil society stakeholder):**‬‬‬‬‬‬‬‬‬‬‬‬‬‬‬‬‬‬‬‬‬‬‬‬‬‬ *If you are publicizing it*, *you must de-identify*.*‬ You have to find a way of de-identifying the data or the information and you have to really press on with education very hard if you are going to do that*. *I think general publication of the maps [is good] without necessarily saying to individuals ‘it is certain vulnerable groups*.*’* *The other thing is–you should know–that certain marginalized groups are not protected even by the law*, *and there is a lot of stigma around them*, *and people still patronize their services*. *So*, *that one is hard to conclude on*. *For instance*, *if you say it is common amongst cab drivers that are driving around [city district]*, *that means you will never call a cab that will be coming from [city district] right*? *That is the risk*.
**Excerpt from interview with P15 (district health management team staff member)** **Interviewer**: *So those [city] maps*, *if you are sitting in that room and you are part of the decision making*, *how would you vote*? *Should it be shared with the public or not*? *Remember there are two maps*: *there is one that shows locations within [city district] and then there is one that shows groups of people in different clusters of those that have this type of TB germ than that one*.*‬*‬‬‬‬‬‬‬‬‬‬‬‬‬‬‬‬‬‬‬‬‬‬‬‬‬‬ **P15**: *It’s a difficult decision*. *It’s a difficult vote because the same considerations have to be taken into account–but more specifically*, *groups of clusters of people is more specific than region*. *So*, *I’d actually go for the region ones*, *so let’s publish the region ones instead of publicizing groups of clusters of people*.*‬* *Because when you look at the map…it doesn’t show numbers*, *it shows dots*. *Dots are identifiers within that specific location so if you are using coordinates*, *that dot is a coordinate*, *it is someone’s home so in essence you are identifying them*. *So*, *I think in terms of saying that ‘we have TB more in [city district]*,*’ say maybe four or 10 cases there–that is quite a big spread out even though it is regionalized*.
**Excerpt from near the end of the second dialogue, showing mixed views on cluster maps:** ***Facilitator***: *Share or not to share about clusters*?*‬‬‬‬‬‬‬‬*‬‬‬‬‬‬‬‬‬‬‬‬‬ ***Male 1***: *Share*! ***Facilitator***: *It’s OK if it’s something in the middle*. ***Male 2***: *We had only two who were left*. ***Female 1***: *You are allowed to sit on the fence*? ***Facilitator***: *Yes*. ***Male 2***: *Share or not*? *Yes or No*! ***Female 3***: *No*! ***Facilitator***: *No*, *too*? ***Male 3***: *No comment*.

In sum, there was no clear agreement on the exact kinds of TB NG-WGS information that should be published in surveillance reports, prevention messaging, or research articles. Views varied widely on issues related to sharing hotspot maps, cluster maps, and data about marginalized subgroups. Participants often recognized complexity involved in making these decisions and indicated the need to develop context-specific best practices, ethical guidelines, and policies about how, when, and in what manner to display and disseminate TB NG-WGS data.

## Discussion

Our results suggest that the scale-up of TB NG-WGS into routine practice in Botswana would be broadly supported by stakeholders there. However, this support was contingent on responsible agencies engaging in extensive planning, stakeholder and community consultation, capacity-building, and ongoing education of the health workforce, public, and patients about the technology. This finding is in-line with other ethics and stakeholder engagement research about TB genomics, which emphasize principles of trust, transparency, and ongoing engagement with professionals and communities by health systems [18–20]. Despite broad and resounding support for the scale-up of TB NG-WGS in Botswana, few advocated for it uncritically or without some reservations. Most were acutely aware of the potential pitfalls of a rushed or under-resourced implementation and gave recommendations about how a scale-up ought to proceed to ensure sustainability and maximized benefits. Notably, participants did not express worries about criminalization stemming from transmission cluster analyses and had relatively few concerns about consent for routine public health re-uses of patients’ data [37, 38], which have both been major issues in global molecular HIV surveillance controversies [25–27]. These findings suggest that ethical frameworks and other guidelines about public health uses of pathogen genomics *in general* are likely to be less useful than ones developed around uses of sequencing for specific pathogens in particular contexts [57]. For example, the ethical stakes in collecting, using, and sharing genomic data about SARS-CoV-2 or *influenza* (generally brief viral respiratory infections) are quite different than doing so for HIV (a lifelong and highly stigmatized infection) or TB (a bacterial infection transmitted through the air that is stigmatized and can be difficult to treat). Further, the social meaning and personal implications of being infected with a specific pathogen differ greatly depending on the context, culture, and socioeconomic situation in which one lives. This makes general ethics frameworks about “pathogen genomics” less preferable than ones written for specific places and diseases. These general insights will be important for stakeholders to keep in mind as applications of pathogen genomics expand [8, 20]. The 2023 WHO rapid communication about using tNGS in TB program makes the need for country-specific guidance for TB sequencing particularly pressing [3].

Some participants discussed distrust in the healthcare system, which is in alignment with previous work on ethical issues in TB NG-WGS [18]. However, more emphasized the need for broad and sustained engagement to cultivate and sustain trust during a rollout. Along with other research [20], these results thus suggest that national TB control programs considering a scale-up of TB NG-WGS should conduct stakeholder engagement starting in the early formative stages. Further, this process should be led by TB survivors, affected people, and local partners. While we tried to model this form of engagement in our study, we mainly spoke with people who work or advocate within Botswana’s TB system in some professional or activist capacity–including survivors-advocates. Future studies could focus more specifically on the views of marginalized people who are disproportionately affected by TB and forms of stigma and discrimination, such as people actively living with HIV/TB coinfection, advanced HIV disease, migrants, members of the indigenous San community, and people living in poverty or crowded housing conditions.

Participants’ responses raised the question of how ongoing stakeholder engagement could become part of the theoretical implementation of NG-WGS for TB in Botswana. We opted for a style of engagement rooted in constructivist frameworks and the identification of areas of consensus and disagreement about ethical issues that shape the implementation of complex technologies. This approach could be made ongoing if NG-WGS for TB were implemented in Botswana and could have value for other settings. However, we do not think that our study represents some kind of ideal form of engagement; rather, it was well-suited to our context. We thus recommend that researchers and health systems studying the acceptability of TB NG-WGS construct plans based on their specific contexts, taking into account local needs, cultures, deliberative traditions, political systems, and situated socio-technical questions pertaining to NG-WGS that might arise in a particular place. For example, as some participants noted, in Botswana, the cultural, social, and political institutions of public *kgotla* meetings (where community-level decisions are discussed) and of *kgosis* (or chiefs) are of great importance. These and other institutions play a central role in the governance of Botswana and the national culture; indeed, they operate alongside liberal-democratic state institutions such as the presidency and parliament (which is advised by a House of Chiefs) and government agencies such as the Ministry of Health and Wellness [54, 56, 58, 59]. These traditional and liberal-democratic state institutions also exist alongside organs of the public sphere that enjoy substantial political freedoms–particularly civil society organizations, news media, non-governmental public awareness-raising campaigns, social media platforms, and others. Therefore, any public engagement around TB NG-WGS in Botswana ought to–as many participants suggested–draw on these and other resources. However, other jurisdictions considering TB NG-WGS implementation will not be operating in similar conditions, ranging from political freedoms to the strength of health systems and public trust in government. Therefore, all TB NG-WGS stakeholder engagement and rollouts should take local realities, institutions, cultures, and ways of knowing into consideration during planning and execution.

For Botswana, our findings show that TB stakeholders are ready for involvement in a NG-WGS scale-up if one moves forward. Further, because there are not standardized models for introducing TB NG-WGS or related technologies such as tNGS into TB control programs in LMICs, a scale-up in Botswana could contribute to developing best practices for other jurisdictions and global health stakeholders [5]. The highest-level takeaway from our study is that while TB stakeholders in Botswana were enthusiastic about a scale-up of NG-WGS for TB, they recognized that such an effort cannot be assumed to be beneficial to TB patients or the country as a whole. Rather, rigorous engagement, capacity-building, planning, education, implementations of other technologies (e.g., GeneXpert), and ethical considerations should inform any decision about how–and indeed whether–to implement it.

## Supporting information

S1 File This is the guide that was used to conduct interviews for the study. (PDF)

S2 File This is the guide that was used to conduct deliberative dialogues for the study. (PDF)

S1 Text This document provides a static link to the video series used in the study. (PDF)

S1 Questionnaire This document provides responses to the PLOS Global Public Health questionnaire on inclusivity in global research. (PDF)
