# Supplementary material for: Botswana tuberculosis (TB) stakeholders broadly support scaling up next-generation whole genome sequencing: Ethical and practical considerations for Botswana and global health
Source: PLOS Glob Public Health. 2023 Nov 15;3(11):e0002479. doi: 10.1371/journal.pgph.0002479 (PMC10651001; doi:10.1371/journal.pgph.0002479)
Supplement: S1 File — (PDF) [file pgph.0002479.s001.pdf]

Interview guide: Botswana TB Bioethics Project

Notes for the Interviewer

[*In each section of the interview guide, use the questions to explore topics related to the key content to be covered in that section.]*

[There is no need to ask every question in each section, and feel free to ask additional questions that are not in the guide, but which are responsive to issues raised by the participant.]

[*Before the interview begins, the interviewee must be consented into the study (including completing the demographic form) and watch the video series before the interview begins.*]

Interview Guide Section:

Section Summary 2
Part 1: Getting introduced to each other and the topic 2
Part 2: Baseline Knowledge 3
Before the participant watches the video series 3
After the participant watches the video series 3
Part 3: The main uses of Next Generation Whole Genome Sequencing 4
Part 4: The potential scale-up of Next Generation Whole Genome Sequencing 5
Section Summary 5
Part 5: Exploring the benefits of Next Generation Whole Genome Sequencing 7
Section Summary 7
Part 6: Exploring the risks of Next Generation Whole Genome Sequencing 8
Section Summary 8
Discussion: Risks of Next Generation Whole Genome Sequencing 8
Part 7: Ending the interview and closing brief-out 9

Key
Text in Yellow: To read out loud to the interviewee
Italics: Question prompts
Bolded text: Questions to ask
Text in red font: Notes for the interviewer
Text within a text box: To be used in case the participant requires an in – depth explanation

Note: TB Community includes TB advocates, TB survivors (Former MDR patients & drug sensitive patients), TB Affected, TB infected, TB Champions, TB Treatment Supporter, & Civil Society

Definition of terms
1. **TB Survivor:** An individual who has had Tuberculosis
2. **TB Affected**: An individual who has had a family member or someone who is close to them who had Tuberculosis
3. **TB Advocate:** An individual who speaks for all those who are affected and/or infected by TB
4. **TB Champion**: An individual who has had TB, and has dedicated their time and resources to TB efforts in the community
5. **TB Celebrity:** An individual who is famous for having had Tuberculosis
6. **TB Infected:** An individual who currently has Tuberculosis disease
7. **TB Treatment Supporter:** An individual who takes on the responsibility of ensuring that someone who is infected TB takes his/her medication as prescribed until they complete treatment.

Part 0: Brief-in

Section Summary

Thank you for agreeing to take part in this study today about TB genomic epidemiology and your views about the potential scale-up of this technology to be part of routine TB disease control in Botswana.
I have turned on the recording for this interview, which will last about an hour. During this interview, I will ask you questions about **Next Generation Whole Genome Sequencing** for TB, to be referred to as TB Sequencing henceforth. We will explore benefits, risks, and other issues related to this technology's general use as well as its potential scale-up to be part of routine TB disease control work.

In this interview, we are going to explore your knowledge and thoughts about TB Sequencing. It is fine if you do not know much about the technology; we will explore the topic as we move along.

There are no right or wrong answers to the questions I will ask you during this interview. The purpose is to learn about your views, thoughts, experiences, and perspectives about TB Sequencing. I am here to learn from you. Also, this is a confidential interview, so you can talk about personal, professional, activist, or community-based views and experiences in our conversation today. My questions are just for guiding our conversation.

- You don't have to answer any of the questions that I ask if you don't want to. You can ask to have a break or end the interview early if you would like, for any reason.

- I will be taking notes as we talk, which will mean that I might be typing at times or writing on a notepad. Please know that I am still listening as I take notes. We're about to start.

- **Did you have any questions about this study before we get started with the interview?**
  - [*Prompt: Respond to any questions. If they are detailed questions about the technology, let the participant know that there will be further chance to discuss those issues as the interview proceeds*.]

- As we move along, I will continue to ask you to speak both generally about this technology and also from your own individual perspective as someone who *is affected by* TB.

- **Speaking specifically from your perspective as part of the TB community, what are your main concerns or questions as we begin this conversation about TB sequencing?**
  - [*Prompt: Remember that there are many unknowns and no wrong answers to many of the questions that I am asking you; we just want to know your perspective.*]

Part 1: Getting introduced to each other and the topic

1. **What is your role in the Botswana TB community, and how would you describe your role?**

2. **How many years have you been active in the TB sector?**
   - *[The interviewer should ask follow-up questions in this section based on what the participant has said up to this point, or just move to the next section]*

Part 2: Baseline Knowledge

***Before* the participant watches the video series**

3. **Had you ever heard of TB sequencing before being approached about this study?**
   - [*Prompt: It is totally fine if the answer is "no." However, if you have heard of COVID-19 variants, that is the same underlying technology as used in TB sequencing.*]

4. **What do you know about the technology?**
   a) **Can you describe some of the uses of TB sequencing?**

You have probably heard about COVID-19 variants. TB Sequencing uses the same underlying technology as the technology used to identify and track the spread of SARS-CoV-2/COVID-19 variants.

5. **What do you know about how COVID-19 variants are identified and tracked?**
   - *[The interviewer should follow the participant's lead, asking follow-up questions]*

(Allow the participants to watch the videos and then continue the discussion)

***After* the participant watches the video series**

You just watched the videos about the fictional Pule family, which shows TB sequencing in action.

6. **What did you think of the videos? Were they easy to follow? Do you have any suggestions on how they could be improved?**
7. **What were some of your key takeaways from the videos, particularly about the role that TB sequencing played?**
   - *[Prompt 1: For example, Mpho was identified as being in a different cluster than her husband Thabo, and she was also diagnosed as having MDR-TB. This was discovered more quickly because of Sequencing]*
   - *[Prompt 2: Recall the video of the Public Health specialists at the Ministry of Health and Wellness, when they debated whether to publish data about hotspots and clusters in Gaborone owing to ethical considerations.]*

   a) ***Do you have any thoughts about the dilemma that they faced?***

Summary

As you learned from the video series that you watched, TB sequencing is a set of tools used by researchers and public health agencies to better understand the TB germ, how TB can develop treatment resistance, how and where TB is transmitted, and many other factors related to TB disease dynamics.

TB sequencing uses the same technology used to identify and track COVID-19 variants, which you have likely heard about in the news.

The main data analyzed in TB sequencing is TB's genetic code: the most basic biological structures of TB. TB genetic data are not human genetic data; they are the genetic data of the TB germs in individuals' sputum samples.

In TB sequencing, researchers compare genetic information about different individuals' TB sputum against one another. Comparing TB sputum samples using these methods allows researchers to see how individuals' TB are different or similar to one another, as well as how TB is evolving. This form of analysis can reveal likely transmission patterns, the presence of drug resistant TB, and a great deal of very detailed information about TB and how it spreads.

Part 3: The main uses of Next Generation Whole Genome Sequencing

[*Identifying drug resistance*]:
One main use of TB sequencing is the rapid identification of drug-resistant TB.

8. **What do you know about drug resistant TB?**
    - [*Prompt: If they don't know, then explain it and solicit their thoughts.*]

TB sequencing can also identify drug resistance much more quickly than methods that are currently used, which require time-intensive culturing processes. Whereas traditional methods take weeks to culture and identify drug resistance, sequencing could reveal this information in days. Once the type of TB germ has been identified, this will help in the following ways:

1. Ensure the person diagnosed with TB is given the appropriate treatment at onset. Drug resistance is treated with different drugs than TB sensitive/susceptible TB treatment
2. Inappropriate treatment will worsen the TB disease, which can occur if there is delay in identifying the resistant drugs
3. Inappropriate treatment can increase TB transmission, as the TB germ continues to increase in the person who is not on the appropriate treatment
4. Inappropriate treatment can lead to death if it is not killing the TB germ
5. Inappropriate treatment can create more resistance e.g., a person with MDR-TB can lead to XDR-TB. (XDR-TB is worse than MDR-TB. XDR-TB is more difficult to treat as there is very limited TB drugs that can treat it)

9. **What are your thoughts about this potential use of TB sequencing?**
    - [*Prompt: it's fine to speculate; we are exploring hypothetical futures, after all*]

[*Identifying transmission patterns*]:
One main use of TB Sequencing is to identify transmission patterns within a population whose samples were sequenced.

- [*Prompt 1: Remember that there are no right or wrong answers; this is about learning your perspective.*]

For example, this can be done to understand TB transmission patterns among people with TB who live in the same city or region or between different types of people by analyzing the biological differences between those people's TB samples.

[*Hotspot mapping*]:
One main use of TB sequencing is to identify and map transmission hotspots in specific areas of a city of region.

Hotspot mapping shows the areas in a city or neighborhood with the most TB cases and high transmission rates, and also how transmission patterns vary from neighborhood-to-neighborhood. Hotspots can be useful to understand which parts of a city or region people have the highest rates of TB infection and TB transmission. Identifying these hotspots, helps health care worker to try and stop or prevent transmission. How; health care workers can help screen for TB, sensitize the community on TB symptom to try and access health services as soon as possible should they have symptoms. Health care workers can provide TB preventative therapy to those with no TB disease in the area. This

10. **What are your thoughts about TB hotspot mapping?**
    - [*Prompt: In the videos, the Public Health specialists at the Ministry of Health and Wellness debated whether to publish hotspot maps due to ethical concerns and issues related to stigma and discrimination.]*

[*Detailed transmission dynamics*]:

One main use of TB sequencing is to determine very detailed transmission dynamics, such as whether people in the same household are in the same transmission network.

11. **What are your thoughts about this use of TB sequencing?**
    - [*Prompt: One benefit of this is to show when people in the same household are actually part of different transmission clusters, which we saw was the case in the videos about the fictional Pule family. This is important since it ensures that every member of the household gets the appropriate treatment, to prevent them from worsening their disease and then transmitting again to the household members. It is usually assumed that people in the same household may have the same disease but it is not always the case, and TB sequencing ensures that we differentiate them*]

TB sequencing can reveal what are sometimes called "hidden" or "cryptic" transmission patterns that traditional methods cannot reveal.

12. **What are your thoughts or reactions in regard to the ability of TB sequencing to reveal hidden transmission patterns, and detailed transmission patterns?**
    - [*Prompt 1: Remember that we are interested in your perspective.*]

While it is not possible to determine who infected whom with 100% certainty using TB sequencing, the technology can be – and is regularly – used to infer (conclude) who was the source of infection in a transmission network. This is a major ethical concern regarding the technology's use.

13. **What are your thoughts about this issue?**
    - [*Prompt: In the videos, the community health workers who investigated Mpho and Thabo's different clusters assured everyone that you cannot tell who infected who using the technology.]*

    a) **Do you think this was an adequate safeguard**? **Give reasons.**
    b) **What else could have been done?**

[*The interviewer should ask follow-up questions in this section based on what the participant has said up to this point, or just move to the next section*]

14. **Speaking specifically from your perspective as part of the TB community, what do you think are the most potentially beneficial uses of TB sequencing?**
    - [*Prompt: Remember that there are many unknowns and no wrong answers to many of the questions that I am asking you; we just want to know your perspective.*]

Part 4: The potential scale-up of Next Generation Whole Genome Sequencing

Section Summary

Ok, now we are going to talk about the potential scale-up of TB sequencing in Botswana. Just a reminder that this is a hypothetical scenario that we are seeking TB stakeholders' views about.

As described in the video series and as we have discussed, the Pules' story takes place in a hypothetical future where sequencing is done routinely in order to inform TB prevention and disease control initiatives.

Currently, TB sequencing is only done in Botswana in a very limited way, as part of research studies. A hypothetical scale-up of the technology into routine public health practice is something that is potentially on the horizon for Botswana and other countries. We want to understand what people who are active in the TB healthcare, advocacy, and policy spaces in Botswana think about this possibility.

Allow me to emphasize again that this is about a hypothetical scale-up of the technology. Currently, there are not active plans that we are aware of to do this; we are exploring a possible future scenario.

One main outcome of a national scale-up of routine TB sequencing in Botswana would be that every single person diagnosed with TB in Botswana would have their TB sequenced and analyzed.

15. **What are your thoughts about this, considering both risks and benefits?**
    - [*Prompt: Consider the main uses of TB sequencing, such as identifying transmission hotspots or clusters that could be targeted for prevention resources.*]
    - [*Prompt: Think back to the videos about the Pule family, particularly to the way that sequencing showed Mpho and Thabo to be in different clusters, and that Mpho had MDR-TB. This is an example of the technology at work.*]

One aspect of the scale-up would be the more rapid (faster) detection of drug-resistant TB, which is more difficult to treat owing to genetic mutations that confer resistance to first-line treatments. Currently, the TB control program can detect drug resistance, but it takes a very long time – weeks. If sequencing was a part of the national TB control program, drug resistant TB could be identified from samples in just a few days.

16. **What are your thoughts about this likely effect of the scale-up of TB Sequencing?**
    - [*Prompt: Remember that there are many unknowns and no wrong answers to many of the questions that I am asking you; we just want to know your perspective.*]

17. **What are some concerns that you would have about the process of the scale-up, to ensure that it was done properly and appropriately?**
    - [*Prompt: For example, public health staff would have to be trained to properly store, process, and analyze the samples*.]
    - [*Prompt: For example, the national TB public health data infrastructure and laboratory infrastructure would have to be greatly expanded.*]

While we do not have exact figure, there would be a lot of monetary costs associated with scaling up TB Sequencing to be part of routine disease control and prevention in Botswana.

18. **What sort of considerations do you think should be taken account of in regard to costs, if TB genomics were to be scaled up?**
    - [*Prompt: Remember that there are many unknowns and no wrong answers to many of the questions that I am asking you; we just want to know your perspective.*]

As you may know, one key aspect of public health practice is that individuals are not provided with the ability to consent or to opt-out of re-uses of their health data for public health purposes. This is done on the basis that the benefits to society of using these data outweigh an individual's right to consent.

19. **Did you know this, and do you think that the introduction of TB sequencing should affect this aspect of public health practice?**
    - [*Prompt: In regard to HIV sequence data, some communities in the USA and others with international patient networks have called for the introduction of consent into public health programs that use HIV sequence data. In asking this question, we are trying to be responsive to stakeholders' concerns; we are not taking a position one way or the other.*]
    - [*Prompt: Remember that there are many unknowns and no wrong answers to many of the questions that I am asking you; we just want to know your perspective.*]

20. **What sort of data protection measures would you want to see in place in the event of a national scale-up of TB sequencing in Botswana?**
    - [*Prompt: Feel free to use your imagination. There would be a huge range of considerations if this technology were to be scaled up, many of which have not yet been considered. We want to hear your perspective.*]

21. **Let's take a step back from the big picture and think about how the scale-up would theoretically affect you. Given your role in the TB community in Botswana, how do you think a scale-up would affect you/your work?**

- [***Prompt:*** ***For example, how would your advocacy work around privacy and confidentiality issues change if TB sequencing became part of routine public health practice in Botswana?***]

22. **What are your thoughts on what the government and other stakeholders should take into account if TB sequencing were to be scaled up to be part of the national TB control programs?**
    - [*Prompt: Remember that there are many unknowns and no wrong answers to many of the questions that I am asking you; we just want to know your perspective.*]

23. **Speaking specifically from your perspective as *part of the* TB community, *what* do you think are some key considerations that should shape a scale-up of TB sequencing in Botswana, if such an initiative were to move forward?**
    - [*Prompt: Remember that there are many unknowns and no wrong answers to many of the questions that I am asking you; we just want to know your perspective.*]

- *[The interviewer should ask follow-up questions in this section based on what the participant has said up to this point, or just move to the next section]*

Part 5: Exploring the benefits of Next Generation Whole Genome Sequencing

Section Summary

Ok, now we are going to talk about the potential benefits of TB sequencing in Botswana.

The video series showed how the analysis of the Pules family' TB sequence data contributed to a successful cluster response investigation that included both of their different transmission networks.

One of the main benefits of TB sequencing is to identify transmission clusters and then target people in those clusters with prevention resources in order to interrupt chains of transmission and thus stop further transmission. This is called "cluster response" and can potentially be done more efficiently and accurately if public health programs have access to TB sequence data.

24. **What are your thoughts about uses of TB sequencing to inform cluster response investigations?**
    - [*Prompt: Remember that there are many unknowns and no wrong answers to many of the questions that I am asking you; we just want to know your perspective.*]
    - [*Prompt: Cluster response that is informed by TB sequencing is not necessarily that much different than standard public health contact tracing investigations; it operates on the same principles, but with the added details provided by TB genomic information.*]

Understanding the distribution of TB disease within a population is a major potential benefit of TB Sequencing. Sequencing can potentially help to identify the presence and spread of drug resistance, track emerging transmission clusters, understand emerging transmission trends, identify neighborhoods where there is transmission activity, and a wide range of other information. This can all help to inform the allocation of resources and overall public health programming.

25. **What are your thoughts about the wide-ranging potential benefits of TB sequencing?**
    - [*Prompt: Remember that this is more about your perspective than about being "right" or "wrong." If you see benefits, feel free to describe them; if you don't, then we can move on.*

TB sequencing can identify drug resistant TB much faster and with greater precision than traditional culturing methods.
Identifying drug resistance can potentially inform diagnosis and treatment of an individual's TB and can also shed light on how drug-resistant TB is spreading or present within a population.

26. **What are your thoughts about this aspect of TB sequencing?**

TB sequencing data that are held by a government ministry could potentially be de-identified and shared with researchers for secondary analyses and publications.
This could help generate detailed insights about the development of TB epidemics, because academic researchers tend to have access to more cutting-edge analytics than public health agencies.

27. **What are your thoughts about this aspect of TB sequencing?**
    - [Prompt: *The use of novel analytics comes with both benefits and risks*.]

28. **Speaking specifically from your perspective as part of the TB community, what do you think about the potential benefits of TB sequencing, in light of the discussion we have had?**
    - [*Prompt: Remember that there are many unknowns and no wrong answers to many of the questions that I am asking you; we just want to know your perspective.*]

- ***[The interviewer should ask follow-up questions in this section based on what the participant has said up to this point, or just move to the next section)***

Part 6: Exploring the risks of Next Generation Whole Genome Sequencing

Section Summary

Ok, in the last section we discussed the potential benefits of TB Sequencing in Botswana. In this section, we will turn to discuss potential risks.

There are risks inherent in any public health intervention, and there are specific risks that come with TB sequencing and analysis.

These risks are related to what the technology can show about disease dynamics, such as transmission patterns and the development of drug resistant TB.

However, risks can also pertain to issues related to how individuals' data are handled and stored by TB disease control professionals. For example, breaches of public health data systems can occur, thus raising concerns about privacy and confidentiality or stigma and discrimination.

We will now discuss some of these risks.

Discussion: Risks of Next Generation Whole Genome Sequencing

One main risk of TB sequencing is that examining the relationships between different people's TB can show detailed information about transmission dynamics, potentially indicating who may have infected who. The technology is regularly used to make inferences about who infected who, but not with 100% certainty. This comes with a range of risks.

29. **What are your thoughts about the potential risks that arise from this using TB sequencing?**
    - [*Prompt: Think back to the videos, when the cluster investigations led Thabo's coworkers to think that he was the original source of the TB outbreak at his work. While that was a rumor that was resolved; if TB sequencing data indicated that Thabo was the likely source of infection and this was found out by his co-workers, that could have affected the outcome.*]

Some of the risks that arise from TB sequencing come from illegitimate or unexpected uses of the data (e.g., from malicious hacks). However, other risks stem from sanctioned uses of the technology to inform epidemiological surveillance and prevention. For example, publishing reports which show that certain areas

of a city are transmission hotspots could stigmatize neighborhoods and people from them by associating them with TB.

30. **What additional risks can you think of in this regard?**
    - [*Prompt: For example, the videos explored ethical issues faced by the Public Health specialists at the Ministry of Health and Wellness in debating whether to publish certain data. Officials would need to make ethical decisions like that regularly if the technology was scaled up.*]
    - [*Prompt: Recall in the videos that the contact tracing investigation for Thabo's contacts involved some rumors circulating about him at work. More precise investigations based on the detailed data that TB sequencing can reveal would potentially lead to more tricky situations in this regard*.]

If TB sequencing was scaled up to be part of routine public health practice in Botswana, it means that people's data would be stored for a longer period (long term). There is a risk that these data could be requested by other entities, such as immigration authorities, courts, or even potential employers.

31. **Would you have any thoughts about the risks of the long-term storage of TB sequence data?**

32. **What could be done to ensure that these data would not be disclosed improperly to third parties?**
    - [*Prompt: Remember that there are many unknowns and no wrong answers to many of the questions that I am asking you; we just want to know your perspective.*]

33. **What are some of the considerations that you would put forward in regard to engaging communities affected by TB prior to a scale-up of TB sequencing to be part of routine public health practice, if that were to move forward?**

34. **How do you think trust in the health system can be maintained or strengthened as technologies like this are implemented?**
    - [*Prompt: For example**, do you think that initiatives like this study, where we are seeking people's views using videos, interviews, and dialogues, to be helpful?***]

While there are many *purported* benefits of TB sequencing, a great deal of these benefits are theoretical. There is not yet enough evidence to say for certain, for example, if cluster response events involving TB sequencing would necessarily be more effective than traditional response methods. However, some argue that the benefits are clear and that they merit making large investments in scaling up TB sequencing infrastructures across the world.

35. **Do you agree with the move to scale up these interventions?**

36. **What kind of evidence would you need to see to persuade you that this was the right thing to do?**
    - *Prompt: Remember that there are many unknowns and no wrong answers to many of the questions that I am asking you; we just want to know your perspective.*]

37. **Speaking specifically from your perspective as part of the *TB* community, *what* do you think about the potential risks of TB sequencing, considering this discussion we have just had?**
    - [*Prompt: Remember that there are many unknowns and no wrong answers to many of the questions that I am asking you; we just want to know your perspective.*]

Part 7: Ending the interview and closing brief-out

38. **Overall, and based on the discussion so far, do you think that there are more benefits or more risks associated with TB sequencing and analysis?**
    a) **Would you support the scale-up of this technology in Botswana? Give reasons**

- *[Prompt 1: There are no right or wrong answers to this question, it is just to get your viewpoint; you can also feel free to speculate. After all, we are talking about a hypothetical future.]*

- Thank you so much for participating in this study. This is the end of our interview today.

39. **Before we finish, is there anything that you would like to say, to close out our conversation, or anything that we didn't cover today?**
    - *[Prompt: You can bring up anything that you would like. For example,* ***did you have any concerns or thoughts about the benefits of the technology that you wanted to discuss further?]***

- Thank you very much again for participating in this study. Have a good day.
    - *[Process the participant incentive and any other matters before saying goodbye.]*
