# Supplementary material for: Botswana tuberculosis (TB) stakeholders broadly support scaling up next-generation whole genome sequencing: Ethical and practical considerations for Botswana and global health
Source: PLOS Glob Public Health. 2023 Nov 15;3(11):e0002479. doi: 10.1371/journal.pgph.0002479 (PMC10651001; doi:10.1371/journal.pgph.0002479)
Supplement: S2 File — (PDF) [file pgph.0002479.s002.pdf]

**Name of Moderator**	
**Date**	
**Group Representation**	
**Location**	
**Length of session**	
**Number of participants**	

Phase A: Norm-setting for deliberative dialogues (~10min) 2
Phase B Facilitator tools for controlling the discussion: 3
Understanding the science 4
Uses of Next – Generation Whole Genome Sequencing in different contexts 4
Overview of Botswana Context (Current) 4
Ethical Challenges associated with NG – WGS as part of TB control 5
Utility of NG – WGS as part of routine public health in Botswana – Scale Up 6
Closing of the dialogue (around-the-room exercise) 6

Phase A: Norm-setting for deliberative dialogues (~10min)

- **[The facilitator takes the group through these group norms to guide discussion; they could also be handed out on a sheet to everyone, projected on a slide, or just walked through verbally depending on the team's preference and the setting of the dialogue.]**

- We will now go through the norms to guide today's discussion.

- If you have any questions as we go through them, please feel free to ask:
[facilitator seeks affirmative "yes" from the group after each item is said]

- We are all here because we care about TB, are affected by TB, and want to end TB in Botswana.

- While we may occupy different social positions or positions of privilege outside of this space, everyone is equal in this space.

- What is said here today is intended specifically for this space, so there is an expectation that we will preserve each other's privacy.
  - However, some of us might want to use the knowledge that we have gained in our work or our advocacy; this is part of the reason for these dialogues.
  - So, if you discuss something that came up today in another context after the dialogue, please do not attribute any statements to a specific person.
  - Does everyone understand this rule? I can explain it again.
[facilitator seeks affirmative "yes" from the group, and answers questions]

- Everyone's voice will be respected, and disagreements or differences of viewpoint will be respectful.

- Everyone here agrees to speak about their views and perspectives during this dialogue. If we are getting near to the end, and you have not spoken, please raise your hand to speak and contribute to the conversation. The facilitator may also call on you.

- Respectful disagreement and expressing differences of viewpoint is encouraged and part of the deliberative process.

- Please raise your hand before speaking; the facilitator will acknowledge you, add you to the queue, and then you can lower your hand. You will then be called upon when it is your turn.

- Just because you raised your hand first does not mean that the facilitator will call on you first, especially if you have already spoken. If you have raised your hand and been placed in the queue, the facilitator will get to you in time.

- The dialogue will proceed in several sections; if you are not called upon in a section, you will retain your place in line for the next section.

- Remember to "step up" to speak, but also to "step back" if you have already spoken. We want as many perspectives as possible, with no one over- or under-represented.

- **[end of norm-setting]**

- Does the group think that we need to add any other norms to guide our discussion and to help make it a constructive collective conversation, or are we ready to move on to the discussion?

- **[facilitator responds to additional norms that the group wants to establish, if any are raised, then moves to the next section]**

Phase B Facilitator tools for controlling the discussion:

[[Each of these should be introduced at the start of the dialogue, so that people can know to expect them during the dialogue.]]

- I will now describe several tools that I may use during the facilitation of the dialogue; you are free to ask any questions about them as I go through them.

- Notecards (three per participant) and pens/pencils have been distributed to everyone; please feel free to write messages down on them and hand them to the facilitator. I may also ask people to stop speaking for a moment and write something down on a card.

- <u>A "pause and reflect" exercise</u>.
  - I may ask everyone to write one thought down a note card and hand it to the facilitator. This is like a "reset button" for the dialogue.

- <u>A "raise your hand if you agree" exercise</u>.
  - If there is a topic that everyone seems to be engaged about or debating, I may re-state the issue and ask people to raise their hand if they agree or disagree.
  - I may then ask for volunteers who agrees to say more, and someone who disagrees to say more about their position.

- <u>I may introduce a "speaking baton" for a period of time</u>
  - When the speaking baton is in use, only the person who is holding it can speak, and the moderators will control who has it.

- Does everyone understand these strategies and tools that I may use to manage our conversation today, and does anyone have any questions at this time?

Understanding the science

1. What is your understanding of Next – Generation Whole Genome Sequencing and the analysis of Next Generation Whole Genome Sequencing Data?
2. What much do you know about how it is used in the prevention & control of infectious diseases?
   [Probes: The videos, COVID-19, HIV, Sources of information/where they learned about it]
3. What is your understanding of how next generation whole genome sequencing, and the analysis of these data compares to the tests we have currently in the public health system in Botswana?
   [Probes: Accuracy, timeliness, identifying drug resistance, determining appropriate treatment, identify people in clusters by comparing the genomes of their TB (such as Mpho and Thabo in the videos)]
4. Do people understand that all of the data uses that we will discuss today in the public health contexts are undertaken without express patient consent?
   [Probes: there is consent for taking the sputum sample at diagnosis, but after that the data uses are without consent; this follows best practices, but are additional concerns about consent present when considering the detailed information that TB WGS data can show?]

Uses of Next – Generation Whole Genome Sequencing in different contexts

5. **TB Diagnosis**
   a. *[General diagnosis van be done very quickly, including potential identification of drug resistance and other factors that WGS data can show]*
6. **TB surveillance**, including publication of reports (involves identifiable and deidentified data)
   a. *[Publication of routine surveillance reports (many potential benefits, some risks)]*
   b. *[Sharing de-identified data with researchers (many potential benefits, some risks)]*
   c. *[All public health surveillance and prevention data uses are done without individual consent]*
7. **TB Contact investigations, Cluster investigations** (reminder: identifiable data)
   a. *[Benefits: more accurate cluster investigations; drawbacks: identifiable data that can show precise transmission relationships/clustering moving through health systems]*
8. **TB treatment & care**
9. **TB prevention and control**
   a. *[More accurate and faster prevention and surveillance, with potential privacy, confidentiality, and consent considerations]*

[Probe; uses at individual, community, and population level]

- *Video reference: In this video, without the sequencing, Thabo and Mpho would likely have been assumed to have been part of the same transmission cluster, but the sequencing clearly showed that they were part of different transmission networks.*
  - *Do people see benefits in this to the health system?*
  - *Do people see benefits in this to the Pule family unit?*
  - *What about to Thabo and Mpho as individuals?*

Overview of Botswana Context (Current)

10. In this section, we will contrast the current Botswana TB control program with how you think TB control processes might be affected by the addition of sequencing and analysis;

I. TB Diagnosis
II. TB surveillance
III. TB Contact investigations, Cluster investigations
IV. TB treatment & care
V. TB prevention and control

Ethical Challenges associated with NG – WGS as part of TB control

11. Trust

I. Intensifying the power wielded by the public health system and its potential to cause new ethical considerations for the national TB program because these tools can reveal very detailed information about transmission dynamics and other factors?

II. **Data sharing;** routine data vs. research
   a. Note that deidentified data can be shared with researchers and that individuals living with TB are not given the ability to consent; this is in-line with best practices, but is this justifiable when using WGS data of TB samples?

III. Data protection
   a. Should there be enhanced data protections that are not currently in place, if whole genome sequencing were to be introduced into the national TB program? Why or why not? What should those protections be? [seek agreement]

IV. Any other ethical issues?

12. Using TB genomic data for public health prevention and surveillance

I. Using hotspot data for prevention and showing hotspot areas to the general public
   - [[Describe what a hotspot is: an area where a lot of transmission is happening or where TB has been identified]]
   - Should hotspot maps be published (e.g., as part of annual reports)?
     - [[Note that keeping the data internal-only is possible, but that there is value in transparency and that the published maps could help others]]
   - Under what circumstances?
   - Possible risks that come with sharing hotpot areas?
     - [[If people have trouble coming up with some, prompt them; emphasize that this could suggest that poor or migrant-dominated areas where TB is likely to be concentrated are dangerous.]]
   - How to maximise the benefits of informing the public while also reducing any potential risks that might arise from sharing this data?

   [Probe: level of sharing; district, town/village, neighbourhood within a town & within the health sector, business sector, health administration etc]

II. Using cluster data in prevention and potentially sharing it with the general public
   - [[Differentiate between hotspot maps and cluster maps: clusters show specific people in transmission clusters in particular areas. They can be used in prevention *in an identified* fashion or published *in a deidentified* fashion.]]
   - Should cluster data be used to *guide prevention outreach* (e.g., to people identified in clusters)?

- Should cluster maps be *published* (e.g., as part of annual reports ***and/or*** by researchers who might be given de-identified TB surveillance data)?
- Under what circumstances?
- Possible risks that come with sharing cluster maps publicly? *Probe on stigmatizing vulnerable populations*.
- How to maximise the benefits of informing the public while also reducing any potential risks that might arise from sharing this data?
- What happens to our understanding of risk if we think about clusters that also highlight other characteristics that TB genomic data can show (e.g., clustered MDR-TB)?

[Probe: level of sharing; district, town/village, neighbourhood within a town & within the health sector, business sector, health administration etc]

Utility of NG – WGS as part of routine public health in Botswana – Scale Up – What is Needed

What is needed to be able to use NG – WGS data as part of day – to - day clinical practice, public health prevention, and surveillance for TB?

- The basic question: How can the *benefits* of TB sequencing and analysis be maximized while minimizing the potential risks or ethical pitfalls?
  - *Note that this is the final substantive section, "if you have something to say, bring it up in the next several minutes; we want to hear from everyone"*

13. Capacity & training needs

I. Public health administrators
II. Health care workers (Lab personnel, Doctors/Nurses, Lab personnel)
III. Patients (how to inform individuals about how data will be used?)
IV. Community (what kind of consultations would be required?)

14. NG – WGS data; clinical and epidemiological data

I. Privacy and confidentiality
II. Managing access, security (including potentially improper data requests)
III. Community engagement
  - Is it necessary? Why or Why not?
  - If yes, in what way?

15. Policy considerations

I. Cost/Benefit analysis: Is it worth it? Why or why not? How could we assess this?
II. Do additional protections need to be written into policy (e.g. limitations on sharing)?
III. Should the issue of consent be revisited in the context of using TB WGS data for public health?

Closing of the dialogue (around-the-room exercise)

- We are now at the end of the dialogue
  - To close our conversation, I will pass the speaking baton to a volunteer. I then ask that volunteer to pass it to his or her left, going around the entire room so that everyone has a chance to say a last word.

    - Please do not respond to others, just say a reflection about something you have taken away from this conversation, addressed to the whole group.

- Thank you everyone for participating. This now concludes today's dialogue.
