# Supplementary material for: Botswana tuberculosis (TB) stakeholders broadly support scaling up next-generation whole genome sequencing: Ethical and practical considerations for Botswana and global health
Source: PLOS Glob Public Health. 2023 Nov 15;3(11):e0002479. doi: 10.1371/journal.pgph.0002479 (PMC10651001; doi:10.1371/journal.pgph.0002479)
Supplement: S1 Text — (PDF) [file pgph.0002479.s003.pdf]

Link to project videos

For Molldrem et al., “Botswana Tuberculosis (TB) Stakeholders Broadly Support Scaling Up Next-Generation Whole Genome Sequencing: Ethical and Practical Considerations for Botswana and Global Health,” *PLOS Global Public Health*.

For any questions regarding the videos or access issues, please email Dr. Stephen Molldrem at stmolldr@utmb.edu or smolldrem@gmail.com.
