# Supplementary material for: Botswana tuberculosis (TB) stakeholders broadly support scaling up next-generation whole genome sequencing: Ethical and practical considerations for Botswana and global health
Source: PLOS Glob Public Health. 2023 Nov 15;3(11):e0002479. doi: 10.1371/journal.pgph.0002479 (PMC10651001; doi:10.1371/journal.pgph.0002479)
Supplement: S1 Questionnaire — (PDF) [file pgph.0002479.s004.pdf]

Inclusivity in global research

PLOS' policy on inclusivity in global research aims to improve transparency in the reporting of research performed outside of researchers' own country or community and ensures that PLOS publications reporting global research adhere to high standards for research ethics and authorship. Authors of relevant research articles may be asked to complete the questionnaire below, which outlines ethical, cultural, and scientific considerations specific to inclusivity in global research. This questionnaire may be requested when researchers have travelled to a different country to conduct research, if research uses samples collected in another country, research with Indigenous populations or their lands, or if research is on cultural artefacts. Researchers travelling to another country solely to use laboratory equipment will not normally be required to complete the questionnaire. However, the questionnaire can be requested at the journal's discretion for any submission – if you have been requested to complete this questionnaire by the PLOS journal you submitted to, please do so.

Please complete the questionnaire below and include this as a Supporting Information file with your manuscript. Note that if your paper is accepted for publication, this checklist will be published with your article in the supporting information files. Please ensure that you reference the checklist in the main body of your manuscript. We suggest adding a subsection 'Inclusivity in global research' to your Methods section and adding the following sentence: "Additional information regarding the ethical, cultural, and scientific considerations specific to inclusivity in global research is included in the Supporting Information (SX Checklist)"

The questions have been designed to be applicable to a wide range of study types, and there are subsections for both human subjects research and non-human subjects research. If any of the questions are not relevant to your research please mark them as "N/A" as appropriate.

Ethical considerations, permits and authorship

This section is applicable to all research types.

Provide details as to who granted permissions and/or consent for the study to take place in the Methods section of your manuscript. This should include the names of **all** ethics boards, governmental organizations, community leaders or other bodies that provided approval for the study. If individuals provided approval refer to these people by their role or title but do not list their name(s).

On page 6, we report approval by the Botswana Health Research and Development Committee:

> "Human subjects research approvals were obtained from the University of California, Irvine Institutional Review Board (IRB), the University of Texas Medical Branch IRB via reliance, and the Botswana Health Research and Development Committee. Participants provided signed consent and were given 50 pulas (~4 USD) per research engagement. Participants could provide consent on forms written in English or Setswana, the official languages spoken in Botswana."

On pages 6-7, we report additional protocols that we followed during recruitment, which involved securing permissions :

> "Participants were Greater Gaborone-based TB stakeholders purposively sampled and recruited from three key groups: (1) TB policy stakeholders (e.g., from non-governmental organizations, district health management teams (DHMTs), and public health officials), (2) TB community stakeholders (e.g., TB survivor-"champions" and other community-based advocates), and (3) TB clinical and research stakeholders (e.g., physicians, nurses, and research staff). Participants were recruited by Victus Global Botswana Organisation (VGBO) staff (CC, SB, and OM) through direct outreach to individuals, organizations, and facilities based on the team's deep contextual knowledge. VGBO is made up of individuals with longstanding ties to the TB care, prevention, and advocacy community in Botswana, and is the host organization for a TB advocacy network. Recruitment outreach included seeking permissions from DHMT offices and related entities, following procedures that are standard in the country, appropriate within Botswana's regulatory frameworks and cultural norms, and part of our approved protocol (34,35)."

If there were any deviations from the study protocol after approval was obtained please provide details of these changes in the Methods section of your manuscript.

There were no protocol deviations after approval was obtained.

Did this study involve local collaborators that are residents of the country where the research was conducted or members of the community studied? If you do not have any authors from said communities, please provide an explanation for this below.

Yes, five of the nine authors are from Botswana, work for the Victus Global Botswana Organisation (VGBO), and live in Greater Gaborone (Sedilame Bagani, Ogopotse Matsiri, Cynthia Caiphus, and Chawangwa Modongo). All recruitment and data collection was led or done by these team members. All authors meet the *PLOS* citeria for authorship.

Everyone listed as an author should meet PLOS' criteria for authorship and all individuals who meet these criteria should be included in the author byline, rather than the acknowledgements. For further information please see the journal's Authorship Policy.

Human subjects research (e.g. health research, medical research, cross-cultural psychology)

Did you obtain written informed consent from a representative of the local community or region before the research took place? How did you establish who speaks for the community? Details of written informed consent obtained from study participants should be reported separately in the Methods section of your manuscript.

We obtained written, signed informed consent from every study participant. We also sought and received signed permission letters from several organizations, particularly District Health Management Team (DHMTs), as part of our recruitment process.

How did members of the local community provide input on the aims of the research investigation, its methodology, and its anticipated outcome(s)?

Through VGBO, members of the local community were involved in every stage of the study process starting from conceptualization (before we wrote the grant) to execution, analysis, and publication. As we describe on pages 7-8:

> "Participants were recruited by Victus Global Botswana Organisation (VGBO) staff (CC, SB, and OM) through direct outreach to individuals, organizations, and facilities based on the team's deep contextual knowledge. VGBO is made up of individuals with longstanding ties to the TB care, prevention, and advocacy community in Botswana, and is the host organization for a TB advocacy network."

As we describe on page 13 of the paper, we also conducted a dissemination session in Gaborone on March 29th, 2023.

When engaging with the local community, how did you ensure that the informed consent documents and other materials could be understood by local stakeholders?

Yes, informed consent documents were available to participants in both English and Setswana, as was the video series we developed. Additionally, interviews and dialogues were conducted by a bilingual team that utilized whichever language participants were most comfortable using (English or Setswana).

Will the findings of the research be made available in an understandable format to stakeholders in the community where the study was conducted (e.g. via a presentation, summary report, copies of publications, etc.)? Please provide details of how this will be achieved.

Yes. In fact, we have already conducted a dissemination session in Gaborone, as we describe on page 13:

> *"The study team also held a dissemination session with TB stakeholders in Gaborone on March 29th, 2023, after data collection had ended, coding had concluded, and initial findings had been workshopped within the team. The half-day event involved sharing findings that were precursors to the themes reported in this paper in a slideshow and handout. The brief presentation was followed by full-group discussion about key issues in the study and participants' thoughts on next steps for TB NG-WGS in Botswana. The conversation involved both English and Setswana discussion, led by SM, SB, OG, BK, and CM. Attendees included participants and other TB and health stakeholders in Botswana. The session was followed by a lunch where conversation continued. While the dissemination session did not involve data collection, feedback from attendees informed ongoing data analysis and future plans."*

Further, publications will be distributed and disseminated to a variety of stakeholders in Botswana as part of the study team's ongoing genomic epidemiology research activities.

Non-human subjects research using specimens/ animals collected as part of the study, or those housed in archival collections. Examples include archaeology, paleontology, botany and zoology.

Did the permission you obtained from a local authority to perform the study include an agreement on access to outputs and benefit sharing? This may include procedures to enable fair distribution of the benefits and resources arising from the research performed. Please include any details of Prior Informed Consent and Benefit Sharing Agreements obtained. These may be required by field-specific regulations, for example the Convention on Biological Diversity (CBD) and the associated Nagoya Protocol.

N/A

If the material used in your study was imported, please A) provide the year it was imported and B) indicate whether permits were obtained to import/export the materials used, C) provide details of any permits obtained. If this information is not available, please indicate this.

N/A

If you used archival specimens, please state how the material used in your study was acquired by the institute it is held in and provide details of any permits obtained for the original excavations/ sample collection. If this information is not available, please indicate this.

N/A

How was the potential cultural significance of the materials collected in your study to local communities considered in your research design? Were Indigenous peoples and/or local researchers and institutions involved with archaeological excavations / collection of specimens? If so, please provide a description of their involvement.

N/A

If your manuscript includes photographs of human remains please indicate whether authors obtained permission from descendants or affiliated cultural communities to do so.

N/A
